# Mitochondrial dysfunction: mechanisms and advances in therapy

**DOI:** 10.1038/s41392-024-01839-8

**Published:** 2024-05-15

**Authors:** Yao Zong, Hao Li, Peng Liao, Long Chen, Yao Pan, Yongqiang Zheng, Changqing Zhang, Delin Liu, Minghao Zheng, Junjie Gao

**Affiliations:** 1 Centre for Orthopaedic Research, Medical School, The University of Western Australia, Nedlands, WA 6009 Australia; 2 Department of Orthopaedics, Shanghai Sixth People’s Hospital Affiliated to Shanghai Jiao Tong University School of Medicine, Shanghai, 200233 China; 3 Institute of Microsurgery on Extremities, and Department of Orthopedic Surgery, Shanghai Sixth People’s Hospital Affiliated to Shanghai Jiao Tong University School of Medicine, Shanghai, 200233 China; 4 State Key Laboratory of Cell Biology, Shanghai Institute of Biochemistry and Cell Biology, CAS Center for Excellence in Molecular Cell Science, Chinese Academy of Sciences, University of Chinese Academy of Sciences, Shanghai, 200031 China; 5 Sixth People’s Hospital Fujian, No. 16, Luoshan Section, Jinguang Road, Luoshan Street, Jinjiang City, Quanzhou, Fujian China

**Keywords:** Cell biology, Diseases

## Abstract

Mitochondria, with their intricate networks of functions and information processing, are pivotal in both health regulation and disease progression. Particularly, mitochondrial dysfunctions are identified in many common pathologies, including cardiovascular diseases, neurodegeneration, metabolic syndrome, and cancer. However, the multifaceted nature and elusive phenotypic threshold of mitochondrial dysfunction complicate our understanding of their contributions to diseases. Nonetheless, these complexities do not prevent mitochondria from being among the most important therapeutic targets. In recent years, strategies targeting mitochondrial dysfunction have continuously emerged and transitioned to clinical trials. Advanced intervention such as using healthy mitochondria to replenish or replace damaged mitochondria, has shown promise in preclinical trials of various diseases. Mitochondrial components, including mtDNA, mitochondria-located microRNA, and associated proteins can be potential therapeutic agents to augment mitochondrial function in immunometabolic diseases and tissue injuries. Here, we review current knowledge of mitochondrial pathophysiology in concrete examples of common diseases. We also summarize current strategies to treat mitochondrial dysfunction from the perspective of dietary supplements and targeted therapies, as well as the clinical translational situation of related pharmacology agents. Finally, this review discusses the innovations and potential applications of mitochondrial transplantation as an advanced and promising treatment.

## Introduction

**Fig. 1:**
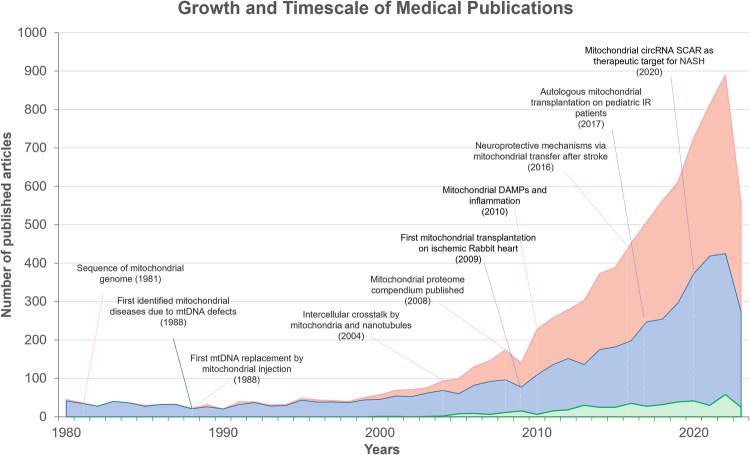
The number of growing published articles or studies from 1980 to Aug 2023, based on mitochondrial medicine (pink), therapies (blue), and clinical trials (green). The publications of therapeutic targets increase following the pathfinding of mitochondria-related molecular mechanisms in many pathologies, including cardiac ischemia/reperfusion (IR) injury, stroke, and nonalcoholic steatohepatitis (NASH). Interventional clinical trials investigating the therapeutic potential of targeting mitochondrial dysfunction are also witnessing a yearly increment discernible in the volume. Data for this figure was extracted from PubMed by searching the term “mitochondri*” in combination with either “medicine”, “transplantation”, “transfer”, “administration”, “delivery”, “restore”, “rescue”, “treatment”, or “therap*”. Data of active clinical trials (recruiting, not yet recruiting, active, not recruiting, completed, enrolling by invitation, unknown status) were acquired from ClinicalTrials.gov

Over the past three decades, mitochondria have emerged as promising therapeutic targets for common diseases, driven by substantial advancements in both mitochondrial biology and clinical research.^1–3^ (See Fig. 1) Evolving from their bacterial ancestor, mitochondria have retained a limited yet unique genomic material known as the mitochondrial DNA (mtDNA), capable of self-replication and encoding indispensable constituents of respiratory complexes located on the inner mitochondrial membrane.^4–6^ Thus, as essential endosymbionts within eukaryotic cells, mitochondria are recognized as the powerhouses orchestrating cellular activities.^7,8^ This central function is well-established on the electrochemical gradient generated by the respiratory chain.^6,9,10^ Mitochondria also play critical roles in mediating lipid metabolism, Ca^2+^ homeostasis, and apoptosis.^2,11^ The concept of mitochondrial dysfunction originates from bioenergetics terminology, which is now extended to a broader relationship corresponding to their cellular environment.^12^ Inherited mitochondrial dysfunction resulting from deficiencies of mtDNA maintenance, and translation, stand as determinants in defining primary mitochondrial diseases (PMD), such as Leigh syndrome, mitochondrial myopathy, encephalopathy, lactic acidosis and stroke-like episodes (MELAS), and chronic progressive external ophthalmoplegia (CPEO).^13–15^ The coexistence of mutant and wild-type mtDNA, referred to as mtDNA heterogeneity, accumulates during aging and age-associated pathogenesis, and is associated with worsened clinical performance.^16^ On the other hand, many common pathologies are convergent to mitochondria, causing secondary mitochondrial dysfunction (SMD), such as heart failure, neurodegeneration, and metabolic syndrome.^17–19^ Abnormal dynamics and quality control, dysfunctional proteostasis, inhibited ATP production, calcium dyshomeostasis, and metabolic reprogramming often simultaneously occur and interplay within pathological conditions, thereby impacting mitochondria in their capacity as biosynthetic and signaling hubs.^1,20^ Consequently, it is unsurprising that mitochondrial dysfunction is frequently implicated in the pathophysiology of various diseases and the aging process.^21–25^

In response to these challenges, advances in mitochondrial biology have spurred the development of mitochondria-targeted therapeutic strategies. There are abundant preclinical studies targeting mitochondrial defects, but clinical studies in humans are still scarce, showing great potential. These range from dietary interventions aimed at counteracting nutritional deficiencies to pharmacological approaches designed to directly modulate mitochondrial dynamics, enhance biogenesis, and mitigate oxidative stress. Notable interventions include: exercise protocols to promote the expression of peroxisome proliferator-activated receptor-gamma coactivator-1 alpha (PGC-1α), dietary supplements to target primary nutrient deficiency, nicotinamide riboside (NR) to augment nicotinamide adenine dinucleotide (NAD) biosynthesis^26,27^, MitoQ for neutralizing mitochondria-derived reactive oxygen species (ROS)^28,29^, the global antioxidant Coenzyme Q10 (CoQ10)^30^, N-acetyl cysteine (NAC)^31^, and the mitochondrial inhibitor ME-344 (known for its anti-tumor properties).^32^. These small molecular drugs can either exert their effects directly on the mitochondria or influence the organelle indirectly by binding to their cytosolic or nuclear regulators.^1^ The strategies of gene replacement therapies and gene editing technologies have also provided hope for addressing the severe prognosis of genetic mitochondrial defect, although the unique mitochondrial genome still entails a more comprehensive mechanistic understanding.^33^

Molecular components from mitochondria, owing to the similarity to their bacterial origins, have long been recognized as potent extracellular signals that elicit immune responses.^34–36^ Recent research into intercellular communication indicated that mitochondria could shuttle across cells horizontally via various transfer mechanisms, a process independent of the vertical inheritance seen during cell division.^37^ Importantly, this paradigm has revealed that mitochondrial components from healthy cells can precipitate a protective response to reconstitute mitochondrial bioenergetics in recipient cells that are metabolically compromised.^38–40^ Additionally, studies also demonstrated that damaged mitochondria were outsourced, as a new form of quality control, to tissue-resided macrophages for clearance in response to oxidative stresses and thus regulating tissue homeostasis.^41,42^ These findings have sparked interest in leveraging transplantation of functionally intact mitochondria and mitochondrial components as therapies for common diseases. Endeavors across various animal models and, even pediatric patients who received central extracorporeal membrane oxygenation (ECMO) support have substantiated the beneficial outcomes of these interventions.^43–46^ However, the clinical translation is still limited, including unstable mitochondrial vitality, inefficient cellular internalization, and transient therapeutic effects, which collectively hinder the medical practice.^47^

Here, as the therapeutic landscape for mitochondrial dysfunction continues to evolve, this review aims to provide a comprehensive view of mitochondrial signaling that controls cell fate, as well as the pathophysiology of mitochondrial dysfunction in common diseases. We also highlight the innovative approaches being pursued to improve clinical management of mitochondrial dysfunction through a critical examination of dietary supplements, pharmacological agents, and mitochondrial transplantation. A deeper understanding of mitochondrial contributions to common pathologies will further elucidate their roles in disease and may reveal co-dependent therapeutic targets.

## Overview of mitochondrial functions and behaviors

**Fig. 2:**
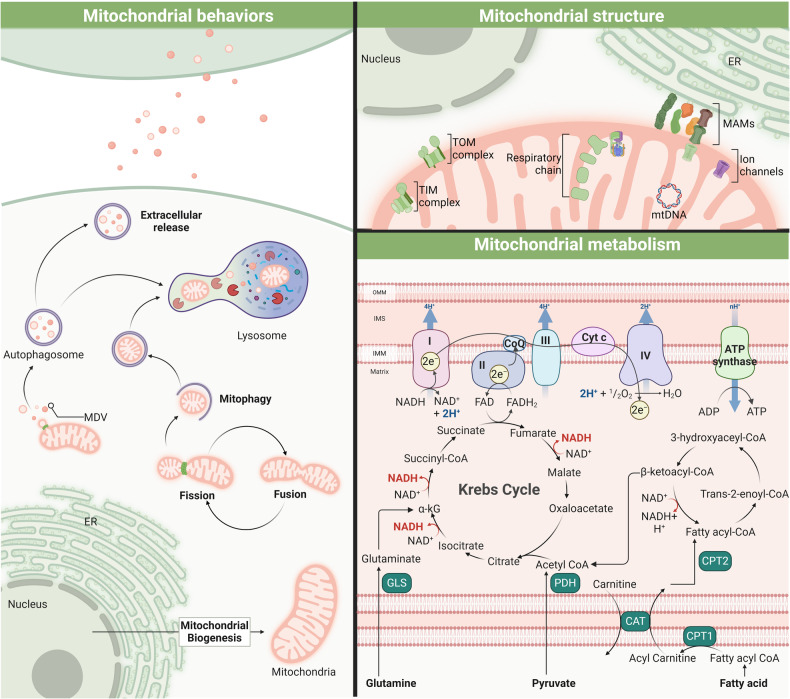
Schematic overview of mitochondrial activities. Mitochondria play a critical role in maintaining cell homeostasis by quality control, energy production, and metabolic regulation. Mitochondrial dynamics, including the processes of fission and fusion, are crucial for shaping mitochondrial structure, ensuring proper distribution across the cell, and facilitating selective clearance of damaged or dysfunctional mitochondria via degradative or secretory pathways, which plays a critical role in mitochondrial quality control. The core of mitochondrial energy production lies in the respiratory chain, fueled by the Krebs cycle and electron transport. The metabolism of fatty acids and glutamine into acetyl-CoA and alpha-ketoglutarate (α-kG), respectively, feeds into the Krebs cycle, culminating in ATP synthesis, illustrating the mitochondria’s central role in cellular energy and metabolic regulation

Mitochondria, the cell’s proverbial powerhouses, present a multifaceted biological landscape in biosynthesis and intra-/inter-cellular signaling pathways. Within these pivotal roles lie a harmonized regulation of static integrity and dynamic processes, such as membrane potential homeostasis and mitophagy, mtDNA maintenance and protein synthesis, inter-organelle contacts and Ca^2+^ regulation, enzymatic activity, and metabolic signaling. Here, we summarize the mitochondrial overview of cell-dependent behaviors, structure basis, and fuel metabolism (See Fig. 2).

In cellular architecture, the mitochondrial network is influenced by quality control systems (biogenesis, dynamics, mitophagy, and proteolysis).^48,49^ The transcriptional activation of mitochondrial biogenesis is governed by synergistic interactions with nucleus. The upregulation of the proliferator-activated receptor-γ co-activator 1α (PGC1α) initiates the transcription cascade, activating transcription factors like nuclear respiratory factors (NRF-1, NRF-2) to the final expression of mitochondrial transcription factor A (TFAM), which promotes mtDNA transcription and replication.^50,51^ These regulatory proteins are often subject to regulation via post-translational modifications (PTMs), exemplified by the action of AMP-activated kinase (AMPK). As an energy sensor, AMPK is allosterically activated by fluctuations in the ATP:ADP or ATP:AMP ratios, which downregulates energy-intensive anabolic pathways while concurrently upregulating energy-generating catabolic processes to restore energetic homeostasis.^52^ In addition, 99% of mitochondrial proteins are encoded by nuclear genes and imported into the mitochondrial compartments through different sorting and import machineries, which are under surveillance by cytosolic ribosomes, the ubiquitin–proteasome system, chaperones, and intramitochondrial proteases.^48,53^ Once synthesized in the cytosol, these precursor proteins, bearing distinctive amino-terminal cleavable targeting motifs, are recognized and translocated by the translocase of the outer/inner membrane (TOM/TIM) complex, such as TOM20, TOM22, TOM40, and TIM23.^54–57^ These channels and transporters are modulated by various PTMs, including phosphorylation, oxidative modifications, ions, and metabolites binding, glycosylation, acetylation, and others. On the other hand, mitochondrial fission and fusion define mitochondrial morphology, distribution, and turnover, which are regulated by highly conserved proteins and organelles to ensure optimal mitochondrial function. Dynamin-related protein 1 (Drp1) facilitates mitochondrial fission by translocating from the cytosol to the outer membrane of mitochondria, where it binds to the adaptors (including MID49, MID51, mitochondrial fission factor) and oligomerizes in a GTPase-dependent fashion to form the fission foci and rings.^58–60^ The depletion of Drp1 and the adaptor proteins leads to the elongation of mitochondria.^61,62^ Preceding the recruitment and assembly of Drp1, the endoplasmic reticulum-mitochondria membrane contact sites were marked in close proximity to future fission sites, contributing to pre-constriction, nucleoid replication, and deep regulation of fission fates.^61,63–65^ Mitophagy is a specialized form of autophagy, dedicated to the selective clearance of aged or damaged mitochondria. This process is tightly regulated by a host of signaling pathways and proteins, most notably the PINK1-Parkin pathway. Upon mitochondrial damage or stress, PINK1 accumulates on the mitochondrial outer membrane, recruiting the E3 ubiquitin ligase Parkin.^66,67^ This subsequently triggers the ubiquitination of mitochondrial proteins, effectively marking these damaged components for removal. These ubiquitinated mitochondria are subsequently engulfed by autophagosomes, which coalesce with lysosomes, facilitating the systematic degradation and recycling of mitochondrial contents. In addition, the mitochondria-derived vesicle represented another form of selective degradation, which might be directed to lysosomes, peroxisomes, or extracellular milieus.^68–71^

Within mitochondria, the folded mitochondrial inner membrane (IMM) provides an extensive surface area of protein import and effective oxidative phosphorylation.^72,73^ Embedded in the IMM, the respiratory chain consists of a series of protein complexes (I to IV) and ATP synthase, collectively forming the core components of mitochondrial energy production. These complexes function sequentially to transfer electrons derived from NADH and FADH_2_ to form an electrochemical gradient. This electron transport culminates in the reduction of molecular oxygen to water. The reduction potential difference driving electron movement through complexes I, III, and IV is used to pump protons, which builds up a protonmotive force (Δp) across the IMM. The resulting electrochemical gradient, or proton motive force, drives ATP synthesis as protons flow back into the mitochondrial matrix through ATP synthase, thereby coupling electron transport with oxidative phosphorylation.^74^ Integral to this process are key metabolic substrates: pyruvate, fatty acid, and glutamine are metabolized to form acetyl-CoA and alpha-ketoglutarate (α-kG), respectively.^75^ These feed into the tricarboxylic acid (TCA) cycle, further fueling electron transport and ATP production.

## Mitochondrial as signaling hubs in defining cell fate

**Fig. 3:**
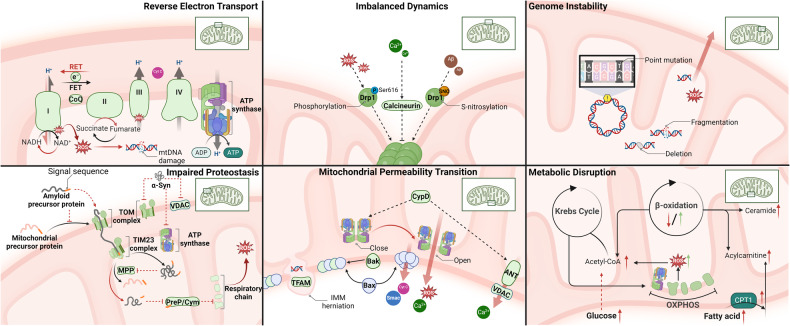
Convergence of cell signaling pathways to mitochondria. This figure delineates the integration of diverse cell signaling pathways converging on mitochondria, illustrating their central role in cellular homeostasis and stress responses. Highlighted pathways include calcium homeostasis, crucial for mitochondrial function and energy production; energy and nutrient sensing through AMPK and mTOR signaling; the innate immune response mediated by cGAS-STING, inflammasomes, and TLR9 endosomal pathways; apoptosis regulation; the unfolded protein response (UPR) as a key element of mitochondrial stress response; and the induction of cellular senescence. Together, these pathways underscore the mitochondria’s pivotal role in orchestrating cellular adaptation and survival mechanisms

Mitochondria epitomize an example of endosymbiotic integration, coordinating the cellular and organelle signal transduction critical for adaptive responses and evolutionary processes. Central to this coordination is the Mitochondrial Information Processing System (MIPS), a sophisticated mechanism that decodes complex amalgamations of ions, proteins, nutrients, and energetic states into targeted genetic programs.^20^ These programs, in turn, initiate a strategic reorganization of metabolic pathways, thereby catalyzing cell proliferation, differentiation, contractility, secretion, and apoptosis, foundational for organismal survival and evolutionary fitness. However, the mechanism by which these pathways intersect within mitochondria to sustain homeostasis is yet to be elucidated. Subsequent discussions will delve into the convergent roles of mitochondria in calcium homeostasis, the intricate networks of energy and nutrient sensing orchestrated by AMPK, mTOR, and Sirtuins, as well as the influence of endogenous mtDNA on innate immune pathways through the cyclic GMP-AMP synthase (cGAS) and stimulator of interferon genes (STING), inflammasomes, and membrane-bound Toll-like receptors (TLRs) signaling. (See Fig. 3) These topics collectively underscore the central role of mitochondria as arbiters of cellular function and integrity, highlighting their potential as therapeutic targets for modulating health and disease states.

### Calcium homeostasis

Calcium ions (Ca^2+^) are critical intracellular second messengers that orchestrate a wide array of cellular functions. Central to this role are mitochondria, which modulate Ca^2+^ signals through sophisticated mechanisms of uptake, storage, and efflux, particularly in response to changes in energy demand, metabolic shifts, and apoptotic signals.^76,77^ This intricate management of calcium allows mitochondria to control the activation of Ca^2+^-sensitive dehydrogenases within the Krebs cycle, subsequently enhancing ATP production to meet cellular energy demands.^78^ Increased mitochondrial Ca^2+^ is beneficial up to a point; however, chronic mitochondrial Ca^2+^ overload triggered Bcl-2-mediated regulated cell death, which is primarily mediated through the induction of the mitochondrial permeability transition pore (mPTP).^79^ In addition, although mitochondrial Ca^2+^ plays a minor role in reducing cytosolic Ca^2+^ concentrations in cardiomyocytes, their function is essential in buffering intracellular calcium levels in non-muscle cells with limited SERCA (sarcoplasmic/endoplasmic reticulum Ca^2+^-ATPase) and NCX (Na^+^/Ca^2+^ exchanger) activity.^80^ In some spatially constrained domains, such as the proximal ER-mitochondria contact sites (also termed ‘mitochondria-associated membranes, MAMs’), calcium release is concentrated, and regulated by PTMs.^81,82^ The structural basis of the calcium release-associated MAMs is a series of tethering complexes that contain both subdomains from ER and mitochondria, such as vesicle-associated membrane protein-associated protein B (VAPB) on the ER and protein tyrosine phosphatase interacting protein 51 (PTPIP51) on the mitochondria (i.e., VAPB-PTPIP51); the inositol triphosphate receptor (IP3R)/glucose-regulated protein 75 (Grp75)/voltage-dependent anion (VDAC1) and sporadic PD-related protein DJ1 complex; mitofusin-2 (Mfn2) on the ER with Mfn1/Mfn2 on the OMM; Rho GTPases Miro1 and Miro2; and others.^83–87^ In aged endothelial cells, the enhancement of MAMs is associated with increased mitochondrial Ca^2+^ uptake but also cell death.^88^

The OMM is highly permeable to Ca^2+^ ions, primarily due to the expression of VDACs which facilitate the direct and indirect calcium flow from the ER, SR, and lysosome.^89,90^ VDACs are differentiated into three isoforms—VDAC1, VDAC2, and VDAC3—each displaying tissue-specific distributions and capable of adopting various voltage-dependent conformations.^91^ Importantly, while VDACs enable Ca^2+^ flux across their open and closed states, the permeability for Ca^2+^ is significantly enhanced in the closed state, where there is concurrently reduced permeability to metabolites.^92^ Furthermore, advanced single cell imaging data indicate that Ca^2+^ may facilitate the VDAC-mediated cation and ATP transport across the OMM.^93^ Additionally, mitochondrial Ca^2+^ uptake is driven by the membrane potential difference (ΔΨ) generated by the activity of the respiratory chain, which is critical for driving the ingress of positively charged ions into the mitochondrial matrix.^94^ To reach the mitochondrial matrix, cytosolic Ca^2+^ has to cross the IMM, mainly through the mitochondrial Ca^2+^ uniporter (MCU) complex. The MCU complex is composed of the channel-forming MCU, the integral scaffolding protein, the essential mitochondrial response element (EMRE), and the mitochondrial calcium uptake (MICU) that senses matrix Ca^2+^ to inhibit or sensitize MCU-mediated Ca^2+^ uptake.^95–98^ Patch-clamp electrophysiology indicated that this inwardly rectifying channel bound Ca^2+^ with extremely high affinity (dissociation constant <2 nM), despite the relatively low cytoplasmic Ca^2+^ concentrations.^99^ However, Ca^2+^ does not remain inside mitochondria but instead is rapidly extruded into the cytoplasm through a complex system of Ca^2+^ antiporters, restoring the basal state. The Na^+^/Ca^2+^ exchanger NCLX localized to the IMM is an established NCX family protein mediating the efflux of matrix mitochondrial calcium.^100^ Deletion of *Slc8b1* (encoding NCLX) in adult mouse hearts causes heart failure and sudden death, correlated with Ca^2+^ overload induced by mPTP.^101^ In addition, the H^+^/Ca^2+^ exchanger, such as leucine zipper, EF hand-containing transmembrane protein 1 (LETM1), leads to mitochondrial Ca^2+^ release in mammalian cells.^102^ The Letm1-deficient mice showed reduced Ca^2+^ mitochondrial uptake at a low cytosolic Ca^2+^ level, with impaired mitochondrial ATP generation capacity, disrupted early embryonic development, altered glucose metabolism, and increased susceptibility to seizure.^103^

### Energy and nutrient sensing

#### AMPK

AMPK is considered the primary sensor of decreased cellular AMP to ATP and ADP to ATP and subsequently acts to increase catabolic reactions and decrease anabolic reactions. Thus, AMPK activity is tightly intertwining its function with mitochondrial health, including biogenesis and dynamics. AMPK relies on PGC-1α to significantly influence GLUT4 and mitochondrial gene expression in skeletal muscle, and it activates PGC-1α through direct phosphorylation at threonine-177 and serine-538.^104^ In addition, AMPK subunit β1 and β2 isoforms knock out mice are physically inactive and have a drastically impaired capacity for treadmill running that is associated with reductions in skeletal muscle mitochondrial content.^105^ Treatment with the AMPK agonist AICAR led to partial correction of COX deficiency in the defective cytochrome c-oxidase (COX) mouse models, and, importantly, significant motor improvement up to normal in knockout/knockin mice for Sco2.^106^ Disruptions in adiponectin and adiponectin receptor 1-induced AMPK-PGC-1α pathway contributed to mitochondrial dysfunction and insulin resistance observed in diabetes.^107^ The underlying mechanism by which AMPK promotes mitochondrial biogenesis is through activation of nuclear respiratory factor-1 (NRF-1).^108^ In subarachnoid hemorrhage (SAH)-induced early brain injury, AMPK and subsequent PGC-1α were activated by exogenous hsp22, thereby maintaining neurological function via the regulation of NRF1/TFAM-dependent mitochondrial biogenesis and Drp1-mediated.^109^ Additionally, mitochondrial quality control is also under the control of AMPK. Direct pharmacological activation of AMPK was sufficient to rapidly promote mitochondrial fission through the mitochondrial fission factor (MFF) phosphorylation, even in the absence of mitochondrial stress.^110^ The AMPK signaling pathway could also activate OPA1‐related mitochondrial fusion and mitophagy, thus attenuating cardiac I/R injury and relieving mitochondrial stress.^111^ Acute exercise-induced mitochondrial oxidative stress initiates AMPK activation, leading to phosphorylated unc-51 like autophagy activating kinase 1 (Ulk1)-dependent mitophagy in skeletal muscle.^112^ This was possibly due to the phosphorylation event of Parkin by ULK1 at Ser108 in its nine amino acid (“ACT”) domain at the early phase of AMPK activation.^113^ Recently, under energy stress conditions, AMPK could translocate to MAMs and directly interact with MFN2 to initiate mitochondrial fission and autophagy.^114^ In addition, in response to the low energy status in mitosis, the energy sensor AMPK phosphorylates and activates MCU, allowing Ca^2+^ entry into mitochondria to boost mitochondrial respiration.^115^ Thus, AMPK emerges as a central regulator of cellular energy balance, directly influencing mitochondrial function and dynamics, with implications for treating metabolic disorders and enhancing mitochondrial resilience against stress and disease.

#### mTOR

Mammalian TOR (target of rapamycin) is a serine/threonine protein kinase in the PI3K-related kinase (PIKK) family that coordinates eukaryotic cell growth, metabolism, and autophagy with environmental inputs, including nutrients and growth factors.^116^ Transcriptionally, mTOR controls the expression of mitochondrial genes (such as *Cytochrome c*, *Atp5*g, and *Cox5a*) by regulating the coactivation of yin-yang 1 (YY1) and PGC-1α.^117^ In addition, mTORC1 controls mitochondrial activity and biogenesis by selectively promoting translation of nucleus-encoded mitochondria-related mRNAs via inhibition of the eukaryotic translation initiation factor 4E (eIF4E)-binding proteins (4EBPs).^118^ On the other hand, mTOR activity is closely related to mitochondrial metabolism.^119^ Metabolomics profiling showed that mTOR independently regulated mitochondrial metabolism in regulatory T cells.^120^ The inhibition of rapamycin-sensitive mTOR complex 1 (mTORC1) also leads to a metabolic shift of mitochondrial respiration to aerobic glycolysis in leukemic cells, possibly in a Bcl-xl-VDAC1-dependent manner.^121^ A recent study reported that FOXK1 suppressed mitochondrial fatty acid oxidation in an mTORC1-dependent manner during the pathogenesis of nonalcoholic fatty liver disease (NAFLD) to non-alcoholic steatohepatitis (NASH).^122^ Additionally, mTORC1 is a regulator of mitochondrial dynamics and cell survival. A study revealed that the nutrient-sensing mTORC1 pathway facilitates DRP1’s pro-fission activity of mitochondria and apoptosis via promoting the translation of mitochondrial fission process 1 (MTFP).^123^ Inhibition of active mTOR sites leads to mitochondrial hyperfusion by reducing MTFP1 translation through the eIF4E-binding protein pathway, showcasing MTFP1’s pivotal role in mTORC1’s modulation of mitochondrial dynamics. In addition to mTORC1, mTORC2 could translocate to MAM and control mitochondrial metabolism via Akt-mediated phosphorylation of the IP3R, Hexokinase 2, and phosphofurin acidic cluster sorting protein 2 (PACS-2).^124^ Conclusively, mTOR orchestrates a complex regulatory network influencing cell growth, metabolism, and autophagy through its impact on mitochondrial gene expression, biogenesis, metabolism, and dynamics.

#### Sirtuin

Sirtuins serve as crucial sensors of the NAD+ to NADH ratio, closely linking them to cellular metabolism, stress response, and longevity. Mitochondrial sirtuins (SIRT3–5) are part of the sirtuin family of NAD^+^-dependent deacylases and ADP-ribosyl transferases, containing an N-terminal mitochondrial signal sequence that dictates the mitochondrial localization.^125,126^ SIRT3 interacts with ATP synthase and is indispensable in mitochondrial membrane potential restoration under mitochondrial homeostasis.^127^ SIRT3 knockout caused decreased levels of superoxide dismutase, catalase, and mitochondrial complex enzymes I/II/III/IV, which exacerbated mitochondrial damage in acute kidney injuries.^128^ Cisplatin-induced decline of SIRT3 also contributed to DRP1-dependent mitochondrial fission in cultured human tubular cells.^129^ However, under mitochondrial stress during cardiac pathology, SIRT3 promoted mitochondrial fusion through the direct deacetylation of OPA1 at the Lys926 and Lys931 residues and thus replenishing the GTPase activity of OPA1 in a NAD^+^-dependent manner.^130^ In addition, an adiponectin receptor agonist promoted AMPK/SIRT3 and increased the protein levels of Mfn2 and OPA1 with no effect on the expression of Drp1, thereby promoting mitochondrial fusion and memory deficits in P301S mice.^131^ SIRT4 could specifically hydrolyze lipoamide cofactors from the DLAT E2 component of the pyruvate dehydrogenase (PDH) complex, thereby inhibiting PDH activity regulation.^132^ SIRT5 supports NADPH homeostasis and antioxidant defense via promoting the desuccinylation and deglutarylation of IDH2 and G6PD, respectively.^133^ Collectively, these insights highlight the intricate roles of mitochondrial sirtuins in modulating mitochondrial function and integrity.

### Innate immunity

Innate immune cells express distinct germ-line-encoded pattern recognition receptors (PRRs) that detect conserved pathogen-associated molecular patterns (PAMPs) unique to microbes.^134^ Alternatively, PRRs could also be activated by the endogenous molecules as dangerous signals called damage-associated molecular patterns (DAMPs) in inflammatory diseases.^135,136^ These molecules include mtDNA, mitochondrial ROS, adenosine triphosphate (ATP), TFAM, cardiolipin, cytochrome c, mitochondrial Ca^2+^, and iron.^137^ Exemplified as mtDNA, mtDNA itself can be recognized by three important PRRs of the innate immune system, cGAS-STING, cytosolic inflammasomes, and TLR9, which triggers type 1 interferon (IFN) responses and/or NF-κB and other signaling pathways to induce proinflammatory cytokines.^135^ Thus, these DAMP-sensing receptors may be valuable targets for therapeutic approaches.

#### cGAS-STING

cGAS (also known as male abnormal 21 domain containing 1 (MB21D1)) is a cytosolic DNA sensor mediating innate immunity. It catalyzes the synthesis of a noncanonical cyclic dinucleotide, 2’,5’ cGAMP, that binds to STING.^138^ Subsequently, cGAS mediates the activation of TANK-binding kinase 1 (TBK1) in Golgi and phosphorylates the transcription factor interferon response factor 3 (IRF3) which then translocates to the nucleus and initiates the transcription of the IFN and ISG.^139^ These have been comprehensively reviewed elsewhere.^140–142^

Compelling evidence underscored the critical role of mtDNA in activating cGAS-STING signaling. Moderate mitochondrial stress, precipitated by TFAM insufficiency, facilitated the translocation of mtDNA into the cytosol, subsequently activating the cGAS-STING-IRF3 signaling cascade.^143^ Such activation is instrumental in engendering a comprehensive antiviral defense mechanism via enhanced expression of a subset of interferon-stimulated genes (ISG). In addition, during the pathogenesis of dengue virus infection, IL-1R-mediated signaling is implicated in the inducement of endogenous mtDNA release, thereby initiating the transcription of IFNB1 and IFNL1 in a paradigm that is unequivocally dependent on the functional integrity of STING and IRF3 pathways.^144^ On the other hand, mtDNA-release-mediated cGAS–STING signaling is involved in the inflammatory activation. STING-deficient mice showed improved inflammation and acute kidney injury (AKI) progression, evidenced by the deactivated mtDNA-cGAS-STING axis and the decreased phosphorylated TBK1 and p65.^145^ Aged microglia affirmed prominent cytosolic accumulation of mtDNA abundance and nucleoids adjacent to the mitochondria outer membrane, which triggered VDAC-dependent cGAS and several type I IFN and proinflammatory genes.^146^ To date, the mechanism of mtDNA release in these settings is not clear. Mechanistically, several studies have reported MOMP is regulated by several pore-forming proteins, such as BAK and BAX, and VDAC. The BAK/BAX is able to facilitate the macropore formation, resulting in MOMP and mtDNA release. This process, however, was directly evidenced by IMM herniation and mitochondrial inner membrane permeabilization (MIMP) rather than mPTP, as the pharmacological inhibitor cyclosporin A (CsA) failed to block the mtDNA and nucleoid release.^147,148^ However, despite the mPTP typically only allows passage of components smaller than 1.5 KDa, mtDNA fragments still can pass through the VDAC oligomer pores and mPTP to activate cGAS-STING.^149,150^ Indeed, the inhibition of mPTP opening with CsA reduced the cytosolic pools of mtDNA by 30%–40%, and VDAC oligomerization inhibition with VBIT-4 reduced cytosolic mtDNA by 50–60%.^151^ Alternatively, the MOMP that mediates mtDNA release and type I IFN response may depend on the level of mitochondrial stress. In moderately stressed mitochondria, mtDNA fragments interacted with the positively charged residues in the N-terminal domain of VDAC1, promoting VDAC1 oligomerization to form pores on the OMM and release mtDNA.^149^ During this procedure, immune recognition of cGAS-STING to mtDNA may be also enhanced by ROS exposure due to the decreased susceptibility to TREX1-mediated degradation.^152^ In addition, a recent study reported that urolithin A-induced mitophagy attenuated cGAS/STING activation by free cytosolic mtDNA, and ameliorated deterioration of neurological function, although the mechanism is unclear.^153^

#### Inflammasome

NLRP3 (Nucleotide-binding domain, Leucine-Rich Repeat, and Pyrin domain-containing protein 3) serves as a multifaceted intracellular sensor, adept at recognizing an extensive spectrum of microbial epitopes, endogenous danger signals, and environmental provocateurs. This recognition precipitates the assembly and activation of a pro-inflammatory signaling platform known as inflammasome. The NLRP3 inflammasome includes an adaptor protein characterized by a caspase activation and recruitment domain (ASC) that harbors two protein interaction domains: an amino-terminal pyrin domain (PYD) and a carboxy-terminal caspase recruitment domain (CARD).^154^ This assembly facilitates the proteolytic cleavage and caspase-1-dependent mobilization of pro-inflammatory cytokines (pro-IL-1β and pro-IL-18), as well as gasdermin D-mediated pyroptotic cellular demise.^154^ Mitochondria are implicated as critical regulators within the NLRP3 inflammasome activation pathway. As an important intracellular signal, ROS mediated the activation of caspase-1 through NLRP3, and thus coactivating the translocation of mtDNA and cytochrome c into the cytosol subject to LPS and ATP stimuli.^155^ Besides, inhibited mitophagy/autophagy leads to mitochondrial ROS generation, prompting the translocation of NLRP3 and its adaptor ASC to mitochondria.^156^ Upon activation by NLRP3 activators, TLR leads to the production of oxidized mtDNA fragments through the IRF1-dependent transcription of CMPK2, a key enzyme in mtDNA synthesis.^157^ This mechanism establishes a feedforward loop that augments inflammation by intertwining NLRP3 activation, ROS generation, and mtDNA release.^135^ In addition to ROS-induced activation, recent metabolomics profiling has shown that the NLRP3 inflammasome activation benefited from ETC integrity through phosphocreatine (PCr)-dependent generation of ATP, despite the existence of unidentified targets within this pathway.^158^ Intriguingly, mitochondrial cardiolipin could bind to NLRP3 and activate inflammasome in a ROS-independent manner.^159^ Additionally, The interrelation of inflammatory pathways with mitochondrial functionality suggests a layered complexity; for example, mtDNA release induced by degenerative retinal pigmented epithelium activated caspase-4- (caspase-11 in mice) and caspase-1-dependent inflammasome, which required cGAS-dependent IFNβ production and gasdermin D–dependent IL-18 secretion.^160^ In a counter-regulatory manner, the antiapoptotic protein Bcl-2 is demonstrated to inversely modulate mitochondrial dysfunction and NLRP3 inflammasome activation, highlighting the intricate balance of mitochondrial engagement in the regulation of inflammatory responses.^161^

#### TLR9

The TLRs constitute a class of type I integral membrane receptors characterized by a conserved structural architecture that encompasses an N-terminal ligand-binding domain, a singular transmembrane helix, and a C-terminal intracellular signaling domain.^162^ These receptors are distinguished by their extracellular domains’ horseshoe-shaped conformation, pivotal for the ligand-induced dimerization that initiates innate immune responses.^163^ TLRs are synthesized within the ER, proceeding through the Golgi apparatus before their distribution to intracellular loci, where they are poised to recognize unmethylated CpG-DNA motifs, notably including mtDNA. The release of mitochondrial DAMPs into the circulation upon cellular injuries activates human polymorphonuclear neutrophils (PMNs) via TLR9, precipitating a sepsis-like systemic inflammatory response syndrome (SIRS).^34^ Additionally, the engagement of the circulating free mtDNA (cf-mtDNA)-TLR9 axis is implicated in the inflammatory progression of NASH, myositis, septic AKI, and chronic restraint stress.^164–167^ Interestingly, mtDNA could shuttle to recipient cells in a PINK1-dependent EV packaging mechanism to promote endosomal trafficking and invasiveness.^168^ The downstream signaling of TLR9 involves the adaptor protein myeloid differentiation primary response protein 88 (MYD88), which activates mitogen-activated protein kinases (MAPKs) and nuclear factor-kappa B (NF-κB), orchestrating an inflammatory cascade.^169,170^ Moreover, mtDNA facilitates endogenous TLR9 activation by translocation into the lysosomal compartment. Intriguingly, mtDNA that escapes autophagy-mediated degradation in cardiomyocytes can initiate TLR9-mediated inflammatory pathways, resulting in myocarditis and dilated cardiomyopathy independent of extracellular mtDNA release.^171^ In the context of podocyte injury within glomerular diseases, TLR9 engagement with endogenously accumulated mtDNA within endolysosomes mediated apoptosis via the p38 MAPK and NFκB signaling pathways, elucidating a complex interplay between mitochondrial components and innate immune activation mechanisms.^172^

### Apoptosis

Mitochondria are indispensable regulators of cell death signaling, playing a central role in intrinsic apoptosis through the dissemination of critical soluble proteins including cytochrome c, Htra2, and Smac after MOMP.^173–175^ The binding of cytochrome c with apoptotic peptidase activating factor 1 (APAF1) engenders the assembly of a pivotal multi-protein complex known as the apoptosome, heralding the initiation of the apoptotic cascade.^176,177^ This apoptotic induction is predicated upon the activation and subsequent oligomerization of pro-apoptotic proteins BAK and BAX, facilitating pore formation and cytochrome c release. Contrastingly, the extrinsic apoptotic pathway intersects with mitochondrial-mediated apoptosis through the caspase-8-facilitated cleavage of BID, a pro-apoptotic BH3-only constituent of the BCL-2 protein family. This enzymatic processing yields a truncated form of BID that robustly instigates MOMP, thereby irreversibly committing the cell to apoptosis. Regulation against MOMP, and consequently apoptosis, is chiefly exerted by the BCL-2 protein family’s anti-apoptotic members, including BCL-2, BCL-W, BCL-X_L, A1, and MCL1, which serve to preserve cell survival by inhibiting pro-apoptotic mechanisms.^178–180^

Intriguingly, it has been observed that the apoptotic machinery renders mitochondrial cGAS immunologically quiescent during apoptosis and subsequent apoptotic clearance.^181,182^ Nonetheless, this regulatory schema is not absolute. Recent empirical findings elucidate that a partial induction of MOMP, orchestrated by BAK/BAX under sublethal apoptotic stress in an APAF1-dependent manner, fails to completely abrogate cGAS–STING signaling.^183^ Instead, it permits the activation of the senescence-associated secretory phenotype (SASP) in senescent cells, indicating a refined interplay between apoptotic signaling pathways, mitochondrial integrity, and immune response modulation. This nuanced interaction underscores the intricate balance maintained by cellular mechanisms between death signaling and immune regulation, providing a fertile ground for further investigative endeavors into the implications of such mechanisms in cellular senescence and disease pathogenesis.

## Mitochondrial dysfunction and related diseases

### Milestone of research history for mitochondrial dysfunction and therapeutics

The historical narrative of mitochondrial exploration has roots extending to the 19th century, a period characterized by the biological paradigm of ‘bioblasts’ that described a cytoplasmic, bacteria-like structure of ubiquitous occurrence, functioning as ‘elementary organisms’. In 1898, Benda termed these structures as ‘mitochondrion’, which evolved from the Greek words, ‘mitos’ (thread) and ‘chondros’ (granules).^184^ Yet, the elucidation of the biochemistry of mitochondria remained silent, that must ‘find a way to make direct chemical analyses of mitochondria’.^185^ A significant breakthrough was achieved in 1934 through Bensley’s pioneering approach of employing differential centrifugation to isolate mitochondria from guinea pig liver.^186^ Subsequent decades were characterized by an elaborate delineation of the morphological and biochemical attributes of isolated mitochondria.^187–191^ An abundance of empirical evidence has demonstrated a compelling linkage between mitochondria and the process of electron transport and oxidative phosphorylation. Importantly, Kennedy’s seminal research revealed the mitochondrial localization of tricarboxylic acid (TCA) cycle intermediates, aerobic oxidation mechanisms of fatty acids, and enzymes implicated in oxidative phosphorylation.^192^ Nevertheless, it was not until 1961 that the elusive electrochemical nature of oxidative phosphorylation was expounded by Peter Mitchell’s ‘chemiosmotic coupling’ theory.^193^ After this revelation, mitochondria regained focus in the late 1990s due to the regulation of cellular apoptosis, particularly through the cytochrome c and Bcl-2 protein family.^194^

The early trajectory of mitochondrial medicine was anchored on the correlation of clinical symptoms with aberrant mitochondrial biochemical signatures and morphological anomalies, as demonstrated in the foundational reports on Luft and Leigh disease.^195,196^ The sequence of the mitochondrial genome in 1981 was a pivotal moment that authenticated the contribution of mtDNA mutations to human pathology.^4^ In the wake of this milestone, the first mitochondrial disorders were characterized, such as Leber’s Hereditary Optic Neuropathy (LHON) due to missense mutations, and Kearns-Sayre Syndrome (KSS) as a result of considerable mtDNA deletions.^197,198^ Subsequent investigations continued to uncover a strong correlation of mitochondrial genetic anomalies with degenerative diseases, aging, and cancer.^199^ This genetic complexity is fueled by diverse mtDNA mutations, intricate phenotypic manifestations, threshold effects, and widespread exceptions. Still, specific mtDNA mutations have been successfully correlated to their clinical phenotypes, including diseases like MELAS, CPEO, and maternally inherited diabetes and deafness (MIDD).^200^ Additionally, nuclear DNA (nDNA) has been elucidated to function directly on the maintenance, translation, and protein synthesis of mtDNA, further convoluting the inheritance patterns of mitochondria.^201^ In 2008, the introduction of a comprehensive mitochondrial proteome inventory - MitoCarta, marked another milestone, and it has now evolved to its 3.0 version.^202,203^

### Pathophysiology of mitochondrial dysfunction in common diseases

**Fig. 4:**
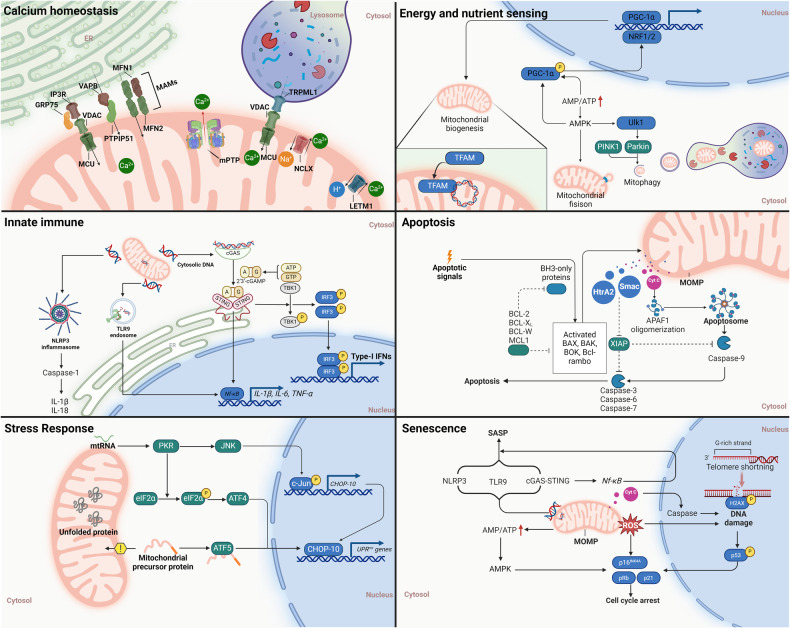
Mechanisms of mitochondrial dysfunction in disease pathogenesis. This figure encapsulates the diverse mechanisms through which mitochondrial dysfunction contributes to common diseases. It illustrates six key dysfunctional processes: 1) Reverse Electron Transport: high Δp fuels the excessive ROS production during ischemia/reperfusion, leading to potential mtDNA damage; the accumulation of misfolded proteins and mistranslated respiratory complexes during AD and PD; imbalanced mitochondrial dynamics, mediated by ROS, Ca^2+^, and Aβ, affect mitochondrial morphology and function; various permeability transition pores lead to mitochondrial cargo release and initiate cell death; disrupted metabolism leading to energy imbalance and exacerbate insulin resistance; and mitochondrial genome instability resulting in altered gene expression and mitochondrial failure. Together, these mechanisms underscore the central role of mitochondria in cellular health and the etiology of various diseases, highlighting potential therapeutic targets for mitigating mitochondrial dysfunction

While the precise molecular mechanisms at play may differ depending on the disease context, common mitochondrial functions and behaviors include compromised bioenergetics, augmented oxidative stress, dysregulated calcium homeostasis, and modifications in mitochondrial dynamics along with quality control mechanisms. (See Fig. 4) In the following sections, we will elaborate on concrete disease examples.

#### Ischemic/reperfusion injuries

Clinically, merely modulating energy balance by decreasing ventricular strain or heart rate might not rectify existing ventricular remodeling.^22^ Therefore, it’s imperative to target mitochondrial dysfunction, given its central role in cardio-cerebrovascular energy depletion and its influence on phenotype enhancement. Ischemia-reperfusion (IR) injury triggers a multifaceted series of metabolic and immune responses that ultimately lead to cell death and tissue remodeling. In the neural context, acute deprivation of oxygen and glucose swiftly impairs neuronal sodium (Na^+^) ion channels, precipitating cell swelling, degeneration, and necrosis.^204^ Concurrently, ischemic conditions activate the hypoxia-inducible factor (HIF), which engages in dual protective and damaging roles. By interacting with the von Hippel-Lindau (VHL) gene product, HIF inhibits proteases, while simultaneously initiating pro-inflammatory and apoptotic pathways via nuclear factor κB (NF-κB). This inflammatory response could exacerbate the hypoxic state by increasing metabolic demand.^205^ Moreover, hypoxia triggers HIF-1-dependent mitophagy, leading to a decrease in metabolic substrates, accumulation of metabolic waste, and dysregulation of calcium homeostasis.^206^ These changes collectively disrupt OXPHOS, diminishing ATP production and compromising mitochondrial permeability transition (MPT).^17,207,208^

Upon reperfusion, the abrupt restoration of blood flow introduces a surge of ROS, the key mediators of acute metabolic disturbance and the subsequent sterile inflammatory response.^204,209^ ROS contributes to cell and tissue impairment through lipid peroxidation within cell membranes and organelles, DNA oxidation, and the activation of matrix metalloproteinases and calpains.^210,211^ In addition, ROS could interact with nitric oxide (NO), fatty acids, or free iron, giving rise to highly reactive molecules such as peroxynitrite, peroxyl radicals, and hydroxyl radicals, which amplify cell death. Given the mitochondria’s role as the primary source of ROS in early reperfusion, their involvement is central to the pathophysiological mechanisms driving tissue injury in IR events, underscoring the critical role of mitochondrial dynamics in the etiology of reperfusion injuries.

#### Reverse electron transport (RET)

RET is a fundamental mechanism in ROS production during IR, involving the backward flow of electrons driven by an elevated proton motive force (Δp) and/or a reduced ubiquinone pool. This reverse flow leads to the incomplete reduction of molecular oxygen, resulting in the formation of reactive intermediates such as superoxide anion (O^2−^), hydrogen peroxide (H_2_O_2_), and the hydroxyl radical (HO•).^212^ In the mitochondrial electron transport chain, 11 potential sites have been delineated where electron leakage may occur, generating superoxide (O^2-^) and/or hydrogen peroxide (H_2_O_2_).^213^ Notably, the IQ site in Complex I and the IIIQo site in Complex III are significantly active, with Complex I identified as the predominant source of superoxide during the reperfusion phase.^17,213^ In the ischemic state, there is a reversal in the function of the succinate dehydrogenase complex (Complex II), which, by utilizing aspartate as a carbon source, increases the biosynthesis of succinate.^17^ This accumulated succinate is then swiftly oxidized within 5 min of reperfusion, sustaining a reduced ubiquinone pool and a high Δp. This scenario sets the stage for electrons to be driven back to Complex I in the reverse direction, thereby facilitating the reduction of NAD+ to NADH and thus promoting superoxide formation. Small molecules targeting suppressors of site IQ electron leak (S1QELs) during RET could reduce ROS production in primary astrocytes, caspase signaling in vivo, and IR injuries in mouse hearts.^214^ The complex I was thus established as the therapeutic target in the early phase of reperfusion, which provided insights to integrate the molecular mechanisms and pathways contributing to IR injury.^215^

#### Ca^2+^ overload and MPT

Under physiological conditions, mitochondria orchestrate calcium dynamics through the interaction with the sarcoplasmic/endoplasmic reticulum (SR/ER), which is crucial for maintaining cardiomyocyte bioenergetics and calcium homeostasis.^216^ During the onset of coronary artery occlusion, regional hypoxia switches mitochondrial OXPHOS to anaerobic metabolism, leading to rapid ATP exhaustion and cardiac contractile failure. This metabolic alteration, accompanied by the decreased function of SERCA and NCLX, perturbates mitochondrial Ca^2+^ homeostasis in the failing heart.^217–219^ When proceeding to the reperfusion phase, SR Ca^2+^ leak via type 2 ryanodine receptor (RyR2) channels triggers mitochondrial Ca^2+^ overload and exacerbates mitochondrial dysfunction and ROS production.^220^ Consequently, intracellular and mitochondrial Ca^2+^ overload activated the mitochondrial chaperone cyclophilin D (CypD), provoking cardiomyocyte death through mitochondrial permeability transition pore (MPTP) opening and excessive cardiac contraction.^221^ Overexpression of the mitochondrial Na^+^/Ca^2+^ exchanger (NCLX) could enhance Ca^2+^ clearance, reduce permeability transition, and prevent myocardial cell necrosis and heart failure due to ischemia. This approach was validated in vivo where ischemic preconditioning coupled with increased NCLX expression reduced infarct size by 43%, highlighting the therapeutic potential of modulating mitochondrial calcium for cardiac protection.^101^

#### Imbalanced mitochondrial dynamics

Mitochondrial fission is integrated by the translocation and binding of fission proteins, which are critical for stress resistance or apoptotic promotion. In adult cardiomyocytes, mitochondria exist as abundant and dispersed round monomers and exhibit low kinetics.^222^ However, their functional integrity is indispensable for cardiac activity, given the heart’s energy-intensive nature. Proper regulation of Drp1 is essential for cell health, illustrating the balance that exists between physiological resistance and apoptosis.^223^ For instance, while the physiological downregulation of Drp1 might be adaptive under specific conditions, its overexpression, especially in pathological states like glucose deprivation, can trigger apoptotic events in cardiomyocytes.^224^ In addition, genetic anomalies in mitochondrial fission and fusion processes can be detrimental during IR, leading to compromised mitophagy. This can, in turn, exacerbate cardiac dysfunction. Notably, there’s a noted association between dysfunctional mitochondrial dynamics and increased vulnerability of cardiomyocytes to IR.^224^ In studies utilizing the ex vivo Langendorff model and the in vivo LAD occlusion model of IR, inhibiting the interaction between Drp1 and Fis1, a key mediator of mitochondrial fission, yielded insightful results. These included decreased mitochondrial fragmentation and reduced release of cytochrome c (cyt c) - a marker of mitochondrial health and initiator of the intrinsic apoptotic pathway. Furthermore, a decline in ROS production was observed, which is crucial given the role of ROS in exacerbating IR injury. The pharmacological inhibition of Drp1-Fis1 interaction not only improved cardiac function but also effectively attenuated apoptosis and autophagy, as evidenced by decreased interactions with caspases and reduced LC3-II levels.^225^

#### Neurodegenerative diseases

Neurodegenerative diseases manifest region- and disease-specific alterations in brain energy metabolism that progress over time.^226^ These metabolic changes are characterized by decreased glucose uptake, impaired tricarboxylic acid (TCA) cycle function, deficiencies in oxidative phosphorylation (OXPHOS), and reduced energetic support provided to neurons by astrocytes and oligodendrocytes. Notably, these perturbations are parallel to mitochondrial dysfunctions, such as genetic defects, kinetics imbalcances, calcium dysregulation, and proteinopathies.^1,227,228^ These mitochondrial pathologies are prevalent across a range of neurodegenerative diseases such as Alzheimer’s disease (AD), Parkinson’s disease (PD), amyotrophic lateral sclerosis (ALS), Huntington’s disease (HD), and frontotemporal dementia (FTD).^229^ Due to the complex mitochondrial dysfunction and multifaced phenotypes of neurodegenerative diseases, it remains challenging to establish whether mitochondrial dysfunctions act as primary causes, consequences, or merely contributing factors in the progression of these diseases. Recent advancements in this field, however, are shedding light on potential underlying mechanisms.

#### Impaired mitochondrial proteostasis

Alzheimer’s Disease (AD), a prototypical neurodegenerative disorder characterized by initial synaptic failure and progressive loss of cholinergic neurons, manifests as memory and cognitive decline.^230–232^ As the hallmarks of AD’s pathology, amyloid-β (Aβ) accumulation and tau aggregation are associated with multifaceted mitochondrial disruption in respiration and dynamics.^233,234^ Aβ protein triggered mitochondrial fission, synaptic loss, and neuronal damage, at least partially via S-nitrosylation of Drp1 to activate GTPase activity.^235^ The binding of Aβ with mitochondria-localized Aβ-binding alcohol dehydrogenase underlies the neurotoxic mechanism, which induces mitochondrial ROS production, cytochrome c release, and ultimate cell death.^236^ On the other hand, the study revealed that Aβ peptides disrupted the turnover of peptides by inhibiting Cym1/PreP, a key mitochondrial metallopeptidase responsible for degrading mitochondrial targeting presequence peptides, resulting in enhanced protein degradation and widespread dysproteostasis in AD.^237^ Concurrently, amyloid precursor protein-derived C-terminal fragments (APP-CTFs) independently impaired mitochondrial morphology and were associated with a Parkin signature of mitophagy failure in human post-mortem AD brains.^238^

The mitochondrial cascade hypothesis, particularly relevant to sporadic and late-onset forms of AD, posits that accumulated oxidative stress reconfigures the cellular response to toxic proteins.^239^ This reconfiguration manifests as age-associated phenotypes driven by deficient cellular clearance mechanisms and disrupted cell cycle processes, highlighting the centrality of mitochondrial integrity in the progression of AD. Experimental models, including transgenic *C. elegans* models expressing human Aβ and tau proteins, have pinpointed defective mitophagy as a key pathological event in AD, suggesting it as a viable target for therapeutic interventions aimed at memory deficits.^240^ Furthermore, mitochondria may offer a protective response against Aβ-induced proteotoxic stress through the mitochondrial unfolded protein response (UPRmt) and enhanced mitophagy, slow the progression of AD, and improve lifespan by stabilizing mitochondrial proteostasis.^241^ Collectively, these insights not only illuminate the multifaceted roles of mitochondria in AD pathology but also suggest novel therapeutic strategies focused on restoring mitochondrial function and enhancing proteostasis.

Parkinson’s Disease (PD) is a progressive neurodegenerative disorder marked by the loss of specific neurons, including dopaminergic cells, where mitochondrial dysfunction frequently emerges as a contributing factor.^242^ This dysfunction is observed across genetic aberrations of the disease, including autosomal dominant cases with SNCA (alpha-synuclein) mutations and copy number variations, and autosomal recessive early-onset forms involving mutations in PARK2 and PARK6 (parkin) genes.^243–246^ Moreover, elevated mtDNA mutations linked to respiratory chain deficiencies are prevalent in neurons particularly vulnerable in PD and in the general process of brain aging, suggesting a possible etiological role of nucleotide pool imbalances and alterations in one-carbon metabolism pathways.^228,247^ The aggregation of alpha-synuclein (α-Syn), a defining feature of PD’s neuropathology through Lewy body formation, further compounds these mitochondrial challenges.^248,249^ Familial and sporadic PD cases often exhibit a dose-dependent mitochondrial accumulation of α-Syn aggregates, leading to the formation of toxic soluble oligomers.^250,251^ These oligomers provoke excessive ROS production and neuronal cell death, with the rate of these deleterious reactions accelerating due to increased oligomer formation and a breakdown in mitochondrial defensive mechanisms.^252^ Though this structural turnover is remarkedly slow due to the kinetic barrier, the reaction speed would sharply increase with enhanced aggregation, formation of β sheet oligomers, and dysregulation of compensatory mechanisms.^252,253^

At the molecular level, mitochondrial membrane constituents such as cardiolipin, ATP synthase, TOM20, and VDAC are known to interact with aggregated α-Syn and thus exacerbating mitochondrial dysfunction.^254–257^ This interaction notably impairs Complex I activity and leads to an overproduction of mitochondrial ROS, which in turn results in reduced ATP production and premature opening of MPTP, leading to neuronal apoptosis.^257^ This apoptotic cascade could potentially be attributed to a compromised protein import machinery caused by disrupted interaction with TOM22.^256^ However, the role of impaired Complex I function as a primary contributor to Parkinsonism is contentious. Conditional knockout of Ndufs4 (subunit of mitochondrial complex I) failed to recapitulate the loss of dopaminergic neurons or the motor deficits throughout the lifespan of mice, suggesting that Complex I dysfunction may not universally lead to parkinsonian symptoms.^258^ Conversely, recent findings from studies using Ndufs2-deficient mice indicate that Complex I dysfunction alone can precipitate a progressive, human-like parkinsonism phenotype, likely due to an early decline in axonal function driven by a shift in mitochondrial metabolic programming.^259^ This nuanced view underscores the complexity of mitochondrial involvement in PD and highlights the potential for therapeutic strategies targeting mitochondrial proteostasis to mitigate disease progression.

#### Aging

Aging is a complex universe that involves organismal ageing and cell senescence. The spectrum of mitochondrial-related aging signatures includes mtDNA mutation and enhanced methylation, genomic instability, decreased mitophagy, and compromised mitochondrial biogenesis.^25,260^ Both mtDNA point mutations and deletions in somatic cells have been shown to accumulate with age, thereby driving energy exhaustion and oxidative damage. In mice with mutated mtDNA polymerase gamma (*Polg*^*D257A/D257A*)^, decreased life span, mitochondrial content, and activity of ETC complexes, as well as apoptotic activation were observed.^261^ Meanwhile, oxidants generated by mitochondria are considered the major source of the oxidative lesions that accumulate with age.^262,263^ In the past, the ‘free radical theory of ageing’ posits that biological aging results from the production of ROS.^264^ A case in point is the effective function of antioxidant enzymes in aging. Superoxide dismutase (SOD2) within the mitochondrial matrix is a critical antioxidant enzyme, mitigating oxidative stress by catalyzing the conversion of superoxide anions (O^2-^) into hydrogen peroxide (H_2_O_2_). In mice model of MnSOD^+/−^ mice and ALDH-2^−/−^ mice (aldehyde dehydrogenase), the assessment of acetylcholine-dependent vascular relaxation reveals a pronounced induction of vascular oxidative stress associated with aging, directly attributable to the compromised antioxidant defense due to partial SOD2 deficiency.^265^ Recent identification of five biomarkers in human plasma that were associated with the senescence-associated secretory phenotype (SASP) and an increased risk of clinical death, involving GDF15、RAGE、VEGFA, PARC, and MMP2.^266^ These hallmarks of biological and metabolic activity decline are often intertwined with mitochondrial dysfunction.^267^ Besides, ROS is also a crucial factor triggering senescence.^268^ In Sod2^−/−^ mice, accumulated senescent cells and impaired complex II activity were observed.^265^ A reduction in mitochondrial ADP sensitivity during aging also increases mitochondrial H_2_O_2_ emission and contributes to age-associated redox stress.^269^ However, studies associated with age-dependent mitochondrial dysfunction engender controversial evidence in identifying the deleterious effect on longevity.^270^ Heterozygous Sod2^+/−^ mice showed increased oxidative stress, as indicated by inactivation of ROS-sensitive enzymes, higher sensitivity to oxidative stress, impaired mitochondrial respiration, and higher levels of DNA oxidative damage in both the nucleus and mitochondria.^271^ Despite this, Sod2^+/−^mice appeared a wild-type lifespan. Recent advancement in understanding the aging process involves the role of apoptosis-induced changes in mitochondrial outer membrane permeability (MOMP), which have been found to facilitate the SASP through the release of pro-inflammatory mtDNA.^183^ This process is initiated by the activation of the pro-apoptotic proteins BAK and BAX, leading to IMM herniation and the subsequent release of mitochondrial matrix constituents, notably mtDNA and TFAM.^147^ Using the small-molecule BAX inhibitor BAI1 to inhibit MOMP could counteract age-associated sterile inflammation and improve lifespan.^183^ Furthermore, the relationship between mitochondrial dysfunction and lifespan is complex, possibly subject to a phenotype threshold or point of irreversibility. Intriguingly, increased oxidative stress resulting from the deletion of sod genes in *C. elegans* does not necessarily shorten lifespan; in some cases, it has been observed to extend it by altering mitochondrial function.^272^ This finding challenges the conventional understanding of oxidative stress and aging. Severe mitochondrial defects and reduced lifespan caused by the global deletion of ubiquinone (UQ) could also be reversed by restoring UQ levels.^273^ Together, these point to an uncoupled link between mitochondrial dysfunction and lifespan.

#### Metabolic syndrome

The indispensable involvement of mitochondria permeates numerous aspects of cellular metabolism, including the processing of diverse substrates like glucose, lipids, and amino acids.^274^ Metabolic syndrome refers to a cluster of metabolic disturbances including obesity, insulin resistance, dyslipidemia, and hypertension, and is associated with complications such as thrombosis, inflammation, cardiovascular disease, and NAFLD.^275–277^ The current evidence base has suggested that aberrations in mitochondrial biogenesis, lipid metabolism, and OXPHOS were involved in the pathogenesis of metabolic syndrome.^277–281^ Furthermore, alterations in mitochondrial dynamics and quality control mechanisms, as well as increased ROS production, contribute to a vicious cycle that exacerbates both mitochondrial dysfunction and metabolic syndrome.^282,283^ Mitochondrial impairments in key tissues such as adipose, skeletal muscle, and liver lead to systemic consequences, including insulin resistance and disrupted lipid homeostasis, which can manifest as components of metabolic syndrome.^284–286^ In light of these findings, targeting mitochondrial pathways may represent a promising therapeutic strategy to mitigate the pathological consequences of metabolic syndrome.

#### Insulin resistance

Insulin-resistant individuals exhibit impaired metabolism or tolerance to glucose challenge through obesity, sedentary lifestyle, high-fat diet, or genetic factors.^276^ Mitochondrial dysfunctions have been intricately linked to the development of insulin resistance, which is a key feature of metabolic syndrome. Metabolomic studies have provided detailed insights into the molecular mechanisms involved in this process. In skeletal muscle, decreased mitochondrial content and/or mitochondrial dysfunction led to a reduction in mitochondrial fatty acid oxidation, along with increased levels of intracellular diacylglycerol (DAG) and acyl CoA.^287^ These fatty acid derivatives activate a serine kinase cascade that augments the phosphorylation of Ser/Thr residues in insulin receptor substrate-1 (IRS-1), resulting in the inhibition of translocation of glucose transporter type 4 and glucose uptake.^288–290^ In the liver, mitochondrial dysfunction exacerbates hepatic insulin resistance and promotes the development of NAFLD by disrupting lipid metabolism, increasing mitochondrial uncoupling, and inducing pro-inflammatory cytokines.^291^ In skeletal muscle, mice lacking malonyl-CoA decarboxylase (MCD) showed reduced fat catabolism and resistance to diet-induced glucose intolerance, indicating a strong connection between skeletal muscle insulin resistance and lipid-induced mitochondrial stress.^292^ Accumulated intermediates in mitochondria from human muscle with insulin resistance, such as acylcarnitine and ceramide, also suggested the insulin resistance-associated stress pathway either by a deficiency in or an overload of mitochondrial oxidative capabilities.^292,293^ Acute and chronic consumption of a high-fat diet significantly enhanced the release of mitochondrial ROS, shifting to a more oxidized state and leading to insulin resistance within skeletal muscle.^294^ In addition, ROS signals could be secondary to respiratory alterations-induced insulin sensitivity when subjected to DAG accumulation, PKCθ activation, and impaired muscle insulin signaling.^295^ These mechanisms suggested that decreased mitochondrial function is regulated by damaging effects caused by nutrient excess and signal impairment.^296^ Moreover, it was also demonstrated that the impact of impaired mitochondrial dynamics and quality control mechanisms, such as mitophagy, on insulin resistance and metabolic syndrome.^283^ Increased Drp1 expression and mitochondrial fission were associated with impaired mitochondrial function, characterized by reduced ATP production and increased ROS generation, which contributed to impaired insulin signaling in skeletal muscle cells.^297^ Conversely, inhibiting Drp1 activity and reducing mitochondrial fission improved mitochondrial function and restored insulin signaling. Collectively, these findings underscore the complex interplay between mitochondrial dysfunction, lipid and amino acid metabolism, and insulin signaling, emphasizing the need for a multifaceted therapeutic approach to address the diverse aspects of metabolic syndrome.

#### Non-alcoholic fatty liver disease (NAFLD)

NAFLD is the most prevalent form of chronic liver disease globally and is closely associated with metabolic syndrome.^298^ This metabolic perturbation in NAFLD is characterized by the abnormal accumulation of carbohydrates and fats in the liver, often driven by processes such as the transformation of excessive fructose intake into triglycerides through de novo lipogenesis.^299^ Additionally, insulin resistance in skeletal muscle and liver also contributes to this metabolic shift towards lipogenesis.^300,301^ As NAFLD progresses to nonalcoholic steatohepatitis (NASH), mitochondria evidence a distinct shift to metabolic maladaptation, indicated by ultrastructural damage, compromised ETC activity, upregulated uncoupling protein content, and increased ROS production.^302^ The uncoupling of OXPHOS and increased ROS generation further compromise mitochondrial ETC function, thereby exacerbating ROS formation. In addition, the hepatocyte mitochondria’s capacity to sense overnutrition may precipitate a reverse cascade of proinflammatory signaling through the release of plasma mtDNA and subsequent activation of the intracellular Toll-like receptor 9 (TLR9) pathway.^164^ Interestingly, in obese rodent models, a decrease in mitochondrial functions, including carnitine palmitoyl-CoA transferase-1 (CPT1) activity, fatty acid oxidation, and cytochrome c protein content, were observed preceding the onset of insulin resistance and NAFLD.^303^ In contrast, in mice fed with an 8-week high-trans-fat high-fructose diet (TFD), changes in mitochondrial energetics and TCA flux lagged behind hepatic insulin resistance.^304^ High-resolution respirometry studies showed that obese human individuals, both with and without NAFLD, exhibit 4.3- to 5.0-fold higher maximal respiration rates of isolated mitochondria compared to lean individuals, yet these rates were 31–40% decreased in NASH individuals.^291^ Recent research also highlighted the role of autophagy-related gene 3 (ATG3) in steatosis during NAFLD progression, with studies indicating that knock-down of hepatic ATG3 could improve fatty acid metabolism and enhance mitochondrial complex activity, partly by reducing c-Jun N-terminal protein kinase 1 activity.^305^

## Therapeutics for mitochondrial dysfunction

### Dietary supplements and diet adjustments

Patients suffering from PMD confront an elevated risk of malnutrition, which is considered as a consequence of metabolic challenges.^306,307^ Similarly, several cellular dysfunctions in SMD associated with mitochondrial pathophysiology-such as escalated catabolic demands, bioenergetic depletion, calcium overload, and oxidative stresses-are known to result in deficiencies in both macro and micro-nutrients.^308,309^ Instances might lead to diminished active nutrient absorption where the disease pathology or therapeutic management involves gastrointestinal obstructions, thereby exacerbating the risk of malnutrition.^310,311^ It is well-established that mitochondrial activity relies heavily on a variety of essential nutrients, including cofactors and antioxidants that maintain bioenergetic production.^308^ Consequently, dietary supplementation strategies devised to counter mitochondrial dysfunction offer diverse nutritional options, including carnitine, CoQ, creatine, and vitamin B2. Carnitine is involved in the transport of long-chain fatty acids to mitochondria, and offers cellular protection by restricting the accretion of these fatty acids through binding with acyl-CoA.^312,313^ Ubiquinone or CoQ facilitates the electron transport from Complexes I and II to Complex III, while concurrently providing antioxidant protection to cell membranes and lipoproteins by suppressing lipid peroxidation.^314^ Creatine, by forming a complex with phosphoric acid, enables the replenishment of ATP, thus serving as an energy buffer reservoir in skeletal muscle.^315^ Riboflavin or Vitamin B2, acting as a cofactor for the electron transport chain, demonstrates the capacity to improve mitochondrial Complex I and II, and enzymatic activity upon supplementation.^316^

Human metabolism harnesses ketone bodies to provide alternative energy to various organs during periods of limited glucose availability.^226,317,318^ These ketone bodies - acetoacetate, β-hydroxybutyrate (βOHB), and acetone - are predominantly synthesized in the liver’s mitochondrial matrix through fatty acid oxidation. The ketogenic diet (KD), characterized by high fat and low carbohydrate content, simulates the metabolic state akin to prolonged fasting. Preclinical studies have highlighted the protective role of both ketogenic diets and exogenous supplementation of ketone body, showing benefits in conditions such as intractable childhood epilepsy, ischemic stroke, AD, PD, MELAS, advanced heart failure, and spinal cord injury.^318–325^ The potential mechanism involves increased ketolysis leading to oxidative stress in mitochondria, which triggers an adaptive cellular response.^317^ This hormetic response is characterized by the activation of key cell-defensive regulators like Nrf2, sirtuins 1 and 3, and AMPK, leading to upregulation of genes and pathways associated with antioxidative and anti-inflammatory responses, enhanced mitochondrial function and biogenesis, DNA repair, autophagy, and reduced anabolic energy expenditure. Notably, decanoic acid, a component of the ketogenic diet, acts as a peroxisome proliferator activator receptor γ (PPARγ) agonist, significantly enhancing mitochondrial citrate synthase and complex I activities in neuronal cell lines (SH‐SY5Y).^326^ However, extended exposure to KD has been linked to cardiac fibrosis in rats, a process involving the activation of *Sirt7*, which in turn inhibited the transcription of genes encoding mitochondrial ribosomes, subsequently impeding mitochondrial biogenesis.^327^

Despite the overall low risk, only a few studies exploring the use of dietary supplementation and structure adjustments have thus far adopted a randomized controlled trial (RCT) design, with most remaining as open-label studies. This is understandable considering the challenges associated with subject recruitment and financial costs due to the heterogeneity of PMD and SMD. Consensus on the clinical management, care, and refined administration of empirical interventions based on dietary supplements is accessible elsewhere.^328^

### Targeted treatment using pharmacological agents

The transition of mitochondria from adaption to a dysfunctional state involves their dual roles: providing energy for cellular and organ function and acting as signal hubs to coordinate global cellular feedback. As the landscape of mitochondrial pathophysiology continues to evolve in common diseases, the spectrum of pharmacological targets for the treatment correspondingly expanded. Several intervention strategies are currently in focus: targeting the redox state of mitochondria, stimulating mitochondrial biogenesis, and modulating mitochondrial dynamics. Pharmacological agents corresponding to these targets are currently under investigation in active and randomized clinical trials (See Table 1). Regardless of the specificities of interventions in patients with PMD and SMD, current therapeutic approaches predominantly favor symptomatic treatment. Small molecule therapies have also progressed significantly, bringing these potential treatments closer to the bedside than ever before. Owing to the clinical heterogeneity of PMDs and SMDs, a panacea remains elusive; thus, the main objective of clinical care continues to be the reduction of morbidity and mortality.

**Table 1:** Current randomized trials of pharmacological agents targeting mitochondrial dysfunction

Drug	Disease	Mode of action	Dose	Enrolment & age	Result	Status	Period	Identifier
CoQ10	PMD^a)^	Antioxidant	10 mg/kg/d (400 mg/d)	24 (12 m– 17 y)	No significant difference in McMaster gross motor function and pediatric quality of life scale	Phase III/Completed	6 months	NCT00432744
	CKD^b)^	Antioxidant	1000 mg/d	26 (30 y–79 y)	Increased free fatty acids and decreased complex medium- and long-chain triglycerides. No significant improvement in VO_2_ peak or total work efficiency	Phase II/Completed	6 weeks	NCT03579693
	ALS^c)^	Antioxidant	1800-2700 mg/d	185 (21 y–85 y)	No significant difference in the ALS Functional Rating Scale-revised (ALSFRSr) Score	Phase II/Completed	9 months	NCT00243932
Ubiquinol MitoQ (MTA)^e)^	PD^d)^	Antioxidant	600 mg/d	11 (40 y–75 y)	No results reported	Phase II/Completed	24 weeks	NCT03061513
	CVD^f)^	Antioxidant	20 mg/d	60 (45 y–75 y)	-	NA/Recruiting	8 weeks	NCT05561556
	CVD	Antioxidant	20 mg/d	112 (>60 y)	-	Phase II/Recruiting	3 months	NCT04851288
	COPD	Antioxidant	NA	24 (>40 y)	-	NA/Recruiting	6 weeks	NCT05605548
	Dilated cardiomyopathy	Antioxidant	40 mg/d	106 (>16 y)	-	Phase II/Recruiting	3 months	NCT05410873
	Heart failure	Antioxidant	40 mg/d	60 (50 y–75 y)	-	NA/Not recruiting	28 days	NCT03586414
	Multiple Sclerosis	Antioxidant	20–40 mg/d	60 (18 y– 70 y)	-	Phase I & II/Recruiting	12 weeks	NCT04267926
SS-31 (MTA)	Friedreich Ataxia	Antioxidant	20–30, 40–60 mg/d	18 (>16 y)	-	Active, not recruiting	52 weeks	NCT05168774
SKQ1 (MTA)	Dry Eye Syndrome	Antioxidant	0.155–1.55 µg/mL	90 ( > 18 y)	Significant improvements in corneal fluorescein staining and lissamine green staining in the central region and lid margin redness, also in the dry eye symptoms	Phase II/Completed	28 days	NCT02121301
	Dry Eye Syndrome	Antioxidant	1.55 µg/mL	452 (>18 y)	Significant impact on clearing of Central Corneal Fluorescein Staining (CCFS) and improvement of Best Corrected Visual Acuity (BCVA) at day 28	Phase III (VISTA-1)/Completed	28 days	NCT03764735
	Dry Eye Syndrome	Antioxidant	1.55 µg/mL	610 (>18 y)	Significant impact on clearing of Central Corneal Fluorescein Staining (CCFS) and improvement of Best Corrected Visual Acuity (BCVA) at day 28	Phase III (VISTA-2)/Completed	28 days	NCT04206020
KH176	PMD	Antioxidant	200 mg/d	20 (>18 y)	No results available	Phase II/Completed	28 days	NCT02909400
	PMD	Antioxidant	NA	24 (0 month - 17 y)	-	Phase II/Recruiting	26 weeks	NCT04846036
	PMD	Antioxidant	100–200 mg/d	27 (>18 y)	No results available	Phase II/Completed	28 days	NCT04165239
	PMD	Antioxidant	800–2000 mg	32 (18 y–55 y)	Well tolerated up to single doses of 800 mg and multiple doses of 400 mg b.i.d. and has a pharmacokinetic profile supportive for a twice daily dosing.	Phase I/Completed	7 days	NCT02544217
Acipimox	Sarcopenia	NAD^+^ boosting	750 mg/d	16 (65 y–75 y)	No results available	NA/Completed	14 days	NCT02792621
	Type 2 Diabetes	NAD^+^ boosting	750 mg/d	31 (40 y–70 y)	Improved insulin sensitivity of type 2 diabetes patients by ∼27% and reduced H_2_O_2_ production by ∼45%, but did not improve basal or insulin-stimulated mitochondrial oxidative capacity	NA/Completed	14 days	NCT00943059
	Obesity	NAD^+^ boosting	750 mg/d	39 (18 y–55 y)	Reduced free fatty acids, improved fasting measures of glucose homeostasis, lipids, and adiponectin. No effect on mitochondrial function, mitochondrial density, or muscle insulin sensitivity	Phase II/Completed	6 months	NCT01488409
	Type 1 Diabetes	NAD^+^ boosting	1000 mg/d	28 (25 y–59 y)	No results available	Phase III/Completed	16 weeks	NCT01816165
NR^g)^	CKD	NAD^+^ boosting	1200 mg/d	26 (30 y–79 y)	Significantly altered TCA cycle intermediates and glutamate and decreased a broad range of lipid groups including triglycerides and ceramides. No significant improvement in VO_2_ peak or total work efficiency	Phase II/Completed	6 weeks	NCT03579693
Metformin	Type 1 Diabetes	Unknown	500–2000 mg/d	23 (20 y–59 y)	Results not available	Phase IV/Completed	6 weeks	NCT01813929
	PMD	Complex I inhibitor	500 mg	61 (>18 y)	Results not available	Phase II/Completed	2 hours	NCT02500628
Idebenone	MELAS Syndrome	Antioxidant, ETC substrate	900–2250 mg/day	27 (8 y–65 y)	Results not available	Phase II/Completed	1 month	NCT00887562
	PPMS^h)^	ETC substrate	10–50 mg/kg/d (2250 mg/d)	85 (18–65 y)	No significant improvement in CSF GDF15 levels	Phase I & II/Completed	3 years	NCT00950248
	Friedreich’s Ataxia	Antioxidant, ETC substrate	900–2250 mg/day	70 (8 y–17 y)	No significant change in the International Cooperative Ataxia Rating Scale (ICARS)	Phase III/Completed	6 months	NCT00537680
Rosiglitazone	HIV Infections	PPAR agonist	8 mg/d	71 (>18 y)	Results not available	Phase II/Completed	48 weeks	NCT00367744
MIN-102	Friedreich Ataxia	PPAR agonist	15 mg/ml	39 (12 y–60 y)	No results available	Phase II/Completed	48 weeks	NCT03917225
REN001	Mitochondrial myopathy	PPAR agonist	NA	213 (>18 y)	-	Phase II/Completed	24 weeks	NCT04535609
Resveratrol	Mitochondrial myopathy	STACs	1000 mg/d	20 (18 y–80 y)	No results available	NA/Completed	20 weeks	NCT03728777
	Aging	STACs	1000–1500 mg/d	59 (>65 y)	Results not available	Phase II/Completed	3 months	NCT02123121
	Type 1 Diabetes	STACs	1000 mg/d	24 (>18 y)	-	Phase I/Recruiting	12 weeks	NCT04449198
	Friedreich Ataxia	STACs	2000 mg/d	25 (>16 y)	-	Active, not recruiting	24 weeks	NCT03933163
Urolithin A	Aging	Fission induction	1000 mg/d	50 (45 y–70 y)	-	NA/Recruiting	28 days	NCT05735886
HU6	Type 2 Diabetes Obesity	Mitochondrial uncoupler	450–600 mg/d	48 (18 y–70 y)	-	Phase II/Not yet recruiting	6 months	NCT06104358

^a)^*PMD* primary mitochondrial disease

^b)^*CKD* chronic kidney disease

^c)^*ALS* amyotrophic lateral sclerosis

^d)^*PD* Parkinson’s disease

^e)^*MTA* mitochondria-targeted agent

^f)^*CVD* cardiovascular disease

^g)^*NR* nicotinamide riboside

^h)^*PPMS* primary progressive multiple sclerosis

#### Preventing mitochondrial stress

As highlighted previously, mitochondria constitute a principal source of ROS. Endogenous enzymatic sources, including transmembrane NADPH oxidases (NOXs) and the mitochondrial electron transport chain (ETC), principally responsible for the production of O_2_^−^ and H_2_O_2_^329,330^. Accordingly, the levels of mitochondrial ROS serve as stress surveillance system for assessing mitochondrial dysfunction and cellular health.^331^ Meanwhile, mitochondria themselves are also targets of persistent oxidative stress, leading to lipid and mtDNA damage or protein posttranslational modifications.^332–334^ Oxidants are also involved in the bidirectional regulation of mitochondrial dynamics, such as mitochondrial cristae and network remodeling. High levels of oxidants can also lead to MPT, further triggering “ROS-induced ROS release (RIRR)”.^335^ Upon exposure to reactive oxygen or nitrogen species, [4Fe-4S]^2+^ clusters in mitochondrial respiratory proteins are oxidized and may result in reversible aconitase inactivation, which impairs the inhibition of ROS production though affecting mitochondrial metabolic flux.^336^ Antioxidants have long been thought to protect mitochondria from damage. Certain compounds specifically target the complex I site, including S1QELs and OP2113, inhibiting RET and ROS production without disturbing normal OXPHOS.^214,337–339^ Quinone-based antioxidants, encompassing CoQ analogs idebenone and EPI-743, alongside a water-soluble Vitamin E derivative sonlicromanol (also known as KH176), are employed as a frontline defense against mitochondrial oxidative damage. These antioxidants exert their protective roles by neutralizing reactive oxygen species and safeguarding the activity of glutathione, a crucial antioxidant within cells^340–342^ Some drugs target MPTP opening and thus reducing RIRR and cell death events, such as classic cyclophilin D (CypD) inhibitor CsA, which occlude the peptidyl-prolyl cis/trans isomerase (PPIase) active site of CypD.^343^ Despite their promising roles, the efficacy of these global antioxidants is constrained by their suboptimal localization within mitochondria.^344^ To overcome this therapeutic hurdle, researchers have turned to lipophilic cations, molecules that inherently possess a low transmembrane activation energy. This characteristic enables them to penetrate the phospholipid bilayer autonomously without necessitating an uptake mechanism, facilitating their accumulation within the mitochondria which boast a high membrane potential (−150 to −170 mV).^345,346^ The advent of this strategy has given rise to mitochondria-targeted antioxidants such as MitoQ and SKQ1.^347,348^ Szeto-Schiller (SS) peptide family (also known as SS-31, MTP-131 or Bendavia) also could directly target cardiolipin peroxidation, independent to mitochondria membrane potential, and inhibit cytochrome c peroxidase and prevent mitochondrial damage.^349^

#### Inducing mitochondrial biogenesis

Mitochondrial biogenesis represents a cellular mechanism of generating new mitochondria, which is instrumental in ensuring tissue homeostasis. Its pharmacological activation emerges as a potent strategy to mitigate diseases characterized by mitochondrial dysfunction.^207^ This multifaceted process comprises a network of therapeutic targets, including mitochondrial energy and nutrient sensors, and downstream effectors such as transcription factors, cofactors, and nuclear receptors (NRs).^344^ Among the molecular entities involved in mitochondrial biogenesis, the PGC-1α acts as a nodal regulator in this intricate network, integrating upstream signals to launch a downstream mitochondrial gene programs facilitating mitochondrial biogenesis.^350^ However, the transcriptional regulation and post-translational modifications of PGC-1α represent intricate molecular events, which introduces substantial challenges in considering PGC-1α as a direct target for pharmacological interventions.^351^

Upstream sensors respond to perturbations in cellular energy status and nutrient availability. For instance, AMPK, as an essential metabolic regulator of glucose and fatty acid, is activated under conditions of energy deprivation, characterized by a high AMP/ATP ratio. This activation triggers mitochondrial biogenesis through the promotion of PGC-1α activity and its translocation into the nucleus.^352^ The NAD^+^-dependent deacetylase sirtuin family, notably SIRT1, plays a significant role in steering mitochondrial biogenesis by deacetylating and activating PGC-1α, in addition to exerting a direct influence on mitochondrial gene expression.^353^ Conversely, the mTORC1, a sensor of nutrient and growth factor availability, exerts a suppressive effect on mitochondrial biogenesis under conditions rich in nutrients.

At the downstream spectrum, an assortment of transcription factors and nuclear receptors react to these upstream signals, orchestrating a concert of nuclear and mitochondrial gene expression to regulate mitochondrial biogenesis. Among these are nuclear respiratory factors 1 and 2 (NRF1 and NRF2), which dictate the transcription of a plethora of nuclear-encoded mitochondrial genes, and peroxisome proliferator-activated receptors (PPARs), governing lipid metabolism and mitochondrial functionality. The estrogen-related receptors (ERRs) also play a crucial role in the synchronization of gene expression necessary for oxidative phosphorylation. Other key players include the cAMP response element-binding protein 1 (CREB1), and forkhead box O (FOXO) transcription factors, which are known to induce PGC-1α transcription. NRs, serving as transcription factors, are also vital constituents in the signaling cascade governing mitochondrial biogenesis. They mediate gene expression in response to lipophilic hormones, vitamins, and dietary lipids, thus playing a role in shaping the mitochondrial biogenic response.

Several pharmacological agents have been identified to exert modulatory effects on these regulatory nodes, thereby enhancing mitochondrial biogenesis. For example, 5-Aminoimidazole-4-carboxamide ribonucleotide (AICAR) and PXL770 act as AMPK activators. AICAR, by mimicking the effects of AMP, stimulates AMPK which subsequently promotes the transcriptional activity of the coactivator PGC-1α, culminating in enhanced mitochondrial biogenesis.^106,354^ Similarly, PXL770 instigates mitochondrial biogenesis by acting as a direct stimulant of AMPK. In a preclinical investigation focusing on Autosomal dominant polycystic kidney disease (ADPKD), PXL770 treatment activates AMPK and restored mitochondrial DNA copy number and PGC-1α mRNA expression in mice with ADPKD.^355^ Another compound, Metformin, widely known for its role in diabetes management, also bolsters mitochondrial biogenesis by activating AMPK, leading to an upsurge in PGC-1α expression and activation.^356^ Furthermore, sirtuin-activating compounds (STACs), such as Resveratrol, enhance activity of the SIRT1-PGC-1α axis, thereby promoting muscle remodeling in Duchenne muscular dystrophy (DMD) mice.^357^ PPAR agonists, such as Bezafibrate and Thiazolidinediones, augment mitochondrial biogenesis by binding to and activating PPARs, which heterodimerize with Retinoid X Receptor (RXR) and bind to PPAR response elements in the DNA, enhancing the transcription of genes involved in mitochondrial biogenesis.^358,359^ Finally, REN001, a synthetic agonist of PPAR-δ, works by preferentially activating PPAR-δ, thereby promoting mitochondrial biogenesis and improving muscle function. These compounds, through their action on specific targets, highlight the potential for strategic pharmacological intervention to enhance mitochondrial biogenesis in the context of mitochondrial dysfunction.

#### Targeting mitochondrial dynamics

Impaired dynamics of mitochondrial fusion and fission have been implicated in numerous prevalent diseases, particularly those featuring neurodegeneration.^360^ The facilitation of mitochondrial fusion is orchestrated by GTPases, notably Mfn1, Mfn2, and Opa1, while mitochondrial fission is mediated by the DRP1.^361^ Mdivi-1, a derivative of quinazolinone, inhibits DRP1-dependent mitochondrial fragmentation and has been demonstrated to confer protection from cardiac IR injury and neurotoxicity.^362,363^ Recently, in vitro experimentation showed that Mdivi-1 partially ameliorated the aberrations in mitochondrial dynamics within human aortic smooth muscle cells (HAoSMCs) derived from bicuspid aortic valve (BAV) patients.^364^ Additionally, in vivo studies on mice with end-stage dilated cardiomyopathy (DCM) revealed that Mdivi-1 inhibited the activation of DRP1 signaling, partially mitigated autophagy inhibition and fatty acid metabolic disorder resultant from low-density lipoprotein receptor-related protein 6 (LRP6) deficiency, and ameliorated cardiac dysfunction.^365^

A critical facet of mitochondrial dynamics is the fragmentation and dynamic clearance of damaged mitochondria. Interestingly, recent findings have suggested that the physiological fragmentation of mitochondria under exercise conditions is an adaptation to energy demands.^366^ Physiological disruption mediated by DRP1 preserves mitochondrial membrane potential and attenuates the expression of factors related to mitophagy. It’s worth noting that inhibiting fission using P110 and Mdivi-1 significantly impairs exercise capacity. Furthermore, emerging evidence suggests that Mdivi-1 may exert effects that are independent of mediating fission, including the reversible inhibition of complex I and RET-mediated ROS production.^367^

#### Uncoupling mitochondrial respiration

OXPHOS is inherently inefficient due to proton leakage across the inner mitochondrial membrane, which facilitates the return of protons to the mitochondrial matrix, bypassing ATP synthase and ATP synthesis. This futile proton cycling, driven by specific respiratory chain complexes and uncontrolled proton permeability, is a crucial modulator of both ROS production and energy expenditure. Pertinently, such dynamics are accentuated in obesity, insulin resistance, and NAFLD. Intriguingly, mild mitochondrial uncoupling can yield therapeutic benefits. The mitochondrial uncoupler 2,4-dinitrophenol (DNP) was initially noted as having potential therapeutic benefits in obesity and metabolic diseases. However, it was ultimately discontinued in clinical practice due to neurological complications and fatalities resulting from overdose.^368^ This led to the development of safer alternatives with higher therapeutic indexes, such as DNP derivatives and controlled-release formulations like DNP-methyl ether (DNP-ME) and controlled-release mitochondrial protonophore (CRMP).^369,370^ The therapeutic mechanism involves reversing insulin resistance in the liver and muscle, associated with reductions in diacylglycerol content and decreased activity of protein kinase C epsilon (PKCε) and PKCθ. Recently, a phase 2a trial evaluated the safety and efficacy of HU6, a metabolic accelerator and mitochondrial uncoupler, in adults with NAFLD and high BMI.^371^ Participants received varying doses from 100 to 400 mg of HU6 or placebo. HU6 showed a significant reduction in liver fat content compared to placebo, with mild to moderate side effects like flushing, diarrhea, and palpitations. This trial highlights the potential of modulating mitochondrial uncoupling as a viable therapeutic approach in metabolic disorders.

### Mitochondrial transplantation-a strategy of organelle replacement

**Fig. 5:**
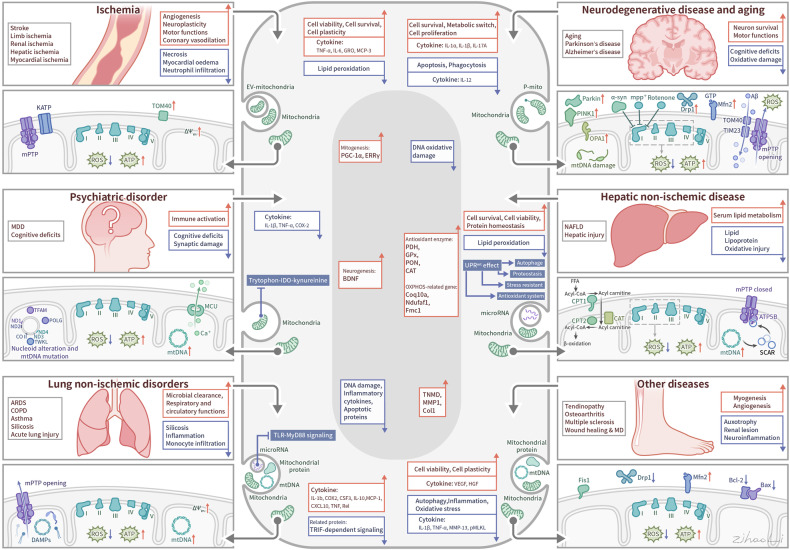
Therapeutic applications of mitochondrial and its component transplantation. This figure concludes therapeutic effects of mitochondria and associated components from different tissues and cells, to the mitochondria level (marked as grey arrows). The increased alterations in boxes are shown as up arrows while decreased alterations in boxes are shown as down arrows. Notably, though tissue level changes can vary, mitochondrial transplantation typically restores ATP production ability and reduces ROS production of damaged mitochondria. Besides, mitochondrial transplantation inhibited the intracellular try-IDO-kyn pathway, thereby improving the cognitive performance of psychiatric disorders and aging. In carbon tetrachloride (CCl_4_)-induced liver injury, transplanted mitochondria can increase a series of anti-oxidative enzymes to improve OXPHOS functions by triggering the UPR^mt^ pathway. On the other hand, MSCs also repress TLR-signaling of macrophages via microRNA transfers to reduce inflammatory responses

Mitochondrial transplantation focusing on functional tissue repair has raised great interest in recent years. Mitochondrial transplantation refers to the artificial complementation or replacement of a dysfunctional mitochondrial network with viable respiratory-competent mitochondria. The basic process involves isolating mitochondrial preparations from healthy tissue and introducing them into the vicinity of the tissue area with mitochondrial dysfunction, either by regional or circulatory injection. Until now, the application list of mitochondrial transplantation has been broadened in various disease models, including IR injuries^47,372–377^, neurodegenerative diseases^378–383^, renal injury^40^, ARDS^384,385^, and cancer^386,387^ (See Table 2 and Fig. 5). Conducting mitochondrial component transplantation not only delineates molecular mechanisms of tissue revitalization but provides possible nodes of intervention in the future.

**Table 2:** Current application of mitochondrial and its component transplantation

Disease Model	Animal	Format	Mitochondrial Source	Methods	Effect	Ref
**Ischemia/reperfusion (I/R)**						
Myocardial ischemia	NZW rabbits^a)^	Mitochondria	Left ventricular	Local injection	**↑**: Postischemic recovery; ↓: Infarct size	^47^
Myocardial ischemia	NZW rabbits	Mitochondria	Pectoralis major muscle	Local injection	↑: Postinfarct cardiac function, angiogenesis, immunomodulation;	^372^
					↓: Myocardial necrosis, apoptosis, and infarction size	
Hepatic ischemia	Wistar rats	Mitochondria	Rat liver	Splenic injection	↓: Tissue insults, oxidative stress, cell death	^375^
MCAO^b)^	Sprague–Dawley rat	Mitochondria	BHK-21 cell	IC & IA injection^c)^	↑: Functional recovery of motor activities, neuron survival; ↓: Brain lesion	^376^
Stroke	C57BL/6 J mice	Mitochondria	Astrocyte	Direct injection	↑: Cell-survival-related signals, plasticity	^43^
MCAO	Sprague–Dawley rat	Mitochondria	Autologous muscle	ICV^d)^ injection	↑: Neurogenesis; ↓: Brain infarct volume, oxidative stress, and cell death	^448^
Warm ischemia	Zucker diabetic fatty rat	Mitochondria	Skeletal muscle	ICA^e)^ infusion	↑: Mitochondrial function; ↓: Infarct size, oedema	^449^
Heart transplantation	C57BL/6 J mice	Mitochondria	Gastrocnemius muscle	ICA infusion	↑: Cold ischemic time; ↓: Graft tissue injury, neutrophil infiltration	^374^
Myocardial ischemia	Yorkshire swine	Mitochondria	Pectoralis major muscle	ICA infusion	↑: Coronary blood flow, myocardial function, perfusion; ↓: Infarct size	^450^
Acute limb ischemia	C57BL/6 J mice	Mitochondria	muscle	Direct injection	↑: Hindlimb function, inflammation; ↓: Infarct size, apoptosis	^377^
Myocardial ischemia	CD1 mice	EVs	iCMs	Direct injection	↑: Mitochondrial biogenesis; ↓: Cardiac remodeling	^390^
Renal I/R injury	C57BL/6 mice	mtDNA, TFAM mRNA, proteins	Mouse MSC	IV^f)^ injection	↓: Renal lesion, inflammation	^451^
Hepatic ischemia	C57BL/6 mice	Mitochondria	hUC-MSC-EVs	IV injection	↓: Hepatocyte apoptosis, proinflammatory cytokines, NET^g)^ formation	^452^
**Neurodegenerative disorders and aging**						
Parkinson’s disease	Sprague–Dawley rat	Mitochondria, P-mito^h)^	PC12, human osteosarcoma	Local injection	↓: Deterioration of dopaminergic neurons, movement disorders	^379^
Parkinson’s disease	C57BL/6 J mice	Mitochondria	HepG2 cell	IV injection	↑: Behavioral symptoms, biochemical assay results	^380^
Parkinson’s disease	Sprague–Dawley rat	Mitochondria, P-mito	Allogeneic liver	IN^i)^ infusion	↑: Rotational and locomotor behavior; ↓: Mitochondrial oxidative damage	^393^
Alzheimer’s disease	C57BL/6 mice	Mitochondria	HeLa cell	ICV injection	↑: Cognitive performance; ↓: Gliosis, neuron loss	^394^
Alzheimer’s disease	NMRI mice	Mitochondria	Allogeneic brain	IN infusion	↑: Spatial memory with Alzheimer’s type degeneration	^395^
Aging	BABL/c mice	Mitochondria	Mice liver	IV injection	↑: Learning and memory ability, muscle function, immune responses	^396^
Aging	Wistar rats	Mitochondria	Rat brain	ICV injection	↑: Mitochondrial function; ↓: Depression like behaviors	^397^
**Psychiatric disorders**						
MDD^j)^	ICR mice	Mitochondria	Hippocampus	IV injection	↑: Antidepressant-like activity; ↓: Neuroinflammation	^381^
Cognitive deficits	C57BL/6 J mice	Mitochondria	Human MSC	IN infusion	↓: White matter and synaptic loss, synaptosomal mitochondrial deficiencies	^400^
**Hepatic non-ischemic disorders**						
NAFLD	C57BL/6 J mice	Mitochondria	HepG2 cells	IV injection	↑: Redox balance; ↓: Lipid content	^405^
Liver injury	C57BL/6 J mice	Mitochondria	HepG2 cells	IV injection	↑: Hepatic function; ↓: Decreased hepatotoxicity	^408^
NASH	C57BL/6 J mice	microRNA	Human liver	IV injection	↓: High Fat Diet-Induced Cirrhosis, insulin resistance, fibrosis	^429^
Liver injury	Kunming mice	Mitochondria	Allogeneic liver	IV injection	↑: Stress resistance, proteostasis; ↓: Oxidation, fibrosis	^407^
**Lung non-ischemic disorders**						
Acute lung injury	C57BL/6 J mice	MSC	Mouse BMSCs	IN infusion	↑: Mouse survival time; ↓: Leukocytosis, albumin leakage	^413^
ARDS	Sprague–Dawley rat	Melatonin–mitochondria	Allogeneic liver	IV injection	↑: Respiratory and circulatory functions; ↓: Oxidative stress, apoptosis	^385^
Silicosis	C57BL/6 J mice	microRNA	Human MSC	IV injection	↑: Bioenergetics; ↓: Monocyte infiltration, inflammation, fibrosis	^41^
COPD	Sprague–Dawley rat	Mitochondria	Airway epithelium	IT^k)^ instillation	↓: Airway hyperresponsiveness to acetylcholine, inflammation	^416^
ARDS	C57BL/6 mice	Mitochondria	Human MSC	IN infusion	↑: Microbial clearance, phagocytosis of alveolar macrophages	^417^
Acute lung injury	C57BL/6 mice	EVs	Human MSC	IN infusion	↓: Inflammatory cell recruitment, lung injury	^418^
Asthma	BALB/c mice	Mitochondria	iPSC-MSC^l)^	IT instillation	↑: Mitochondrial function; ↓: Asthma inflammation	^415^
Acute lung injury	C57BL/6 mice	mtDNA and proteins	Human adipose MSC	IV injection	↑: Macrophage mitochondrial function; ↓: Lung inflammation and injury	^384^
**Central nerve injuries**						
Spinal cord injury	Sprague–Dawley rat	Mitochondria	BHK Cells	Local injection	**↑**: Rat neurobehaviors, nerve electrophysiology, muscle activities;	^420^
					↓: Oxidative stress and the secondary inflammatory response	
Spinal cord injury	Sprague–Dawley rat	Mitochondria	bilateral soleus muscles	Local injection	**↑**: Sensory and locomotor functions;	^421^
					↓: Demyelination, oxidative stress, mitochondrial fragmentation, apoptosis	
Spinal cord injury	Sprague–Dawley rat	Mitochondria	Rat BMSCs	Direct injection	**↑**: Locomotor function recovery	^422^
Spinal cord injury	C57BL/6 mice	P-mito	BMDMs^m)^	IV injection	**↑**: Lower limb motor function, nerve innervation, macrophage repair;	^423^
					↓: mitochondrial dysfunction and proinflammatory profile of macrophage	
**Other diseases**						
Multiple sclerosis	C57BL/6 mice	EVs	Neuro stem cell	ICV injection	↓: Auxotrophy, neuroinflammation	^425^
Tendinopathy	Sprague–Dawley rat	Mitochondria	L6 rat myoblast cell	Local injection	↓: Inflammation, apoptosis	^426^
Wound and muscle dystrophy	C57BL/6, sgca^null^ mice	Mitochondria	Human platelet	Direct injection	↑: Pro-angiogenic function, myogenesis	^424^
Osteoarthritis	Male Wistar rats	Mitochondria	L6 rat myoblast cell	Direct injection	↑: Bone surface, bone volume; ↓: Joint destruction, autophagy	^427^

^a)^*NZW* New Zealand White

^b)^*MCAO* middle cerebral artery occlusion

^c)^*IA* intra-artery; *IC* Intracerebral

^d)^*ICV* intracerebroventricular

^e)^*ICA* intracoronary artery

^f)^*IV* intravenous

^g)^*NET* neutrophil extracellular traps

^h)^*P-mito* Peptide-mediated mitochondria

^i)^*IN* intranasal

^j)^*MDD* Major depressive disorder

^k)^*IT* intratracheal instillation

^l)^*iPSCs* induced-pluripotent stem cells

^m)^*BMDMs* bone marrow-derived macrophages

#### Ischemia/reperfusion

Intervention targeting disrupted mitochondria at the early stage of reperfusion was proven to be effective in reducing subsequent cell necrosis.^47,374,388^ This was possibly due to prevention of the ROS burst derived from RET that induced reversible modification of Cys39 on the ND3 subunit of complex I.^215,389^ To reverse mitochondrial damage induced by ischemia, pioneering work of mitochondrial transplantation was conducted by McCully, J. D. et al. who directly applied autologous mitochondria into the infarction zone on hearts of New Zealand White rabbits instantly before reperfusion. Besides enhanced postischemic myocardial function and decreased ROS, data from proteomics also revealed that receiving exogenous mitochondria compensated for the impaired respiration and energy generation capability of myocytes.^372^ In addition, higher expression of non-mitochondrial cargos from donor induced pluripotent stem cell-derived cardiomyocytes (iCMs), such as PGC-1α and ERRγ mRNA, also facilitated mitochondrial biogenesis in cardiac IR injuries.^390^ The transplanted components upregulated mitochondrial ETC protein expression in a PGC-1α-dependent manner. In the rat livers and brains, mitochondrial transplantation also attenuated cell deaths and oxidative response caused by IR.^375,376^ Notably, it was evidenced that improvements in ventricular function could be achieved by mitochondrial transplantation in pediatric patients, though clinical trials in humans require further investigation.^46^

#### Neurodegenerative diseases and aging

Mitochondrial energetic disruption, oxidative injuries, and dynamic abnormalities are vital features of aging-related neurodegenerative diseases and traumatic injuries.^18,226,391,392^ Recently, mitochondrial-targeting therapy has revealed its potential in postponing the pathological course of PD. In a rat PD model induced by 6-hydroxydopamine (6-OHDA), both allogeneic and xenogeneic mitochondrial injection to the medial forebrain bundle (MFB) reversed the movement disorder and attenuated the deterioration of dopaminergic neurons.^379^ A similar strategy showed improvement of behavioral symptoms in PD mice after intravenous administration of HepG2 cell-derived mitochondria.^380^ Intranasal delivery of mitochondria to 6-OHDA-lesioned rats showed significant improvement in rotational and locomotor behavior, dopaminergic neuron survival, and oxidative damage, demonstrating potential for bypassing the blood-brain barrier and promoting neuronal recovery.^393^ In addition, hippocampal mitochondria showed neuroprotective and antidepressant effects in neuroinflammatory astrocytes and microglia.^381^ Transfer of active intact mitochondria into AD mice also significantly improved cognitive performance, decreased neuronal loss, ameliorated mitochondrial dysfunction in the brain and liver, without observed toxicity, suggesting a novel therapeutic approach for AD.^394,395^ In aging models, injecting mitochondria from young animals into aged ones has been shown to improve cognitive, motor, and depression-like behaviors, enhance mitochondrial function, and modulate metabolic pathways, presenting a promising approach for combating aging and age-related mood disorders.^396,397^

#### Psychiatric disorders

Multiple mechanisms of psychiatric disorders involve cerebral oxygen metabolic changes. Due to their important role in redox balance, it is possible for mitochondria to play a role in energy metabolism in psychiatric disorders.^398,399^ Attenuation of mitochondrial oxidative stress and structural damage were favorable in improving psychiatric symptoms, including depression, anxiety, and other cognitive deficits.^381,397,400^ In these models, ROS level and mitochondrial morphology were normalized via mitochondrial transplantation. In addition, BDNF signal transduction is vital to the regulation of depressive-like behaviors.^401^ Exogenous mitochondria promote BDNF-related neurogenesis in major depressive disorder (MDD), improving anti-depressant effects. Neuronal protection-related gene expression, such as that of Nfe2l1, was also upregulated after mitochondrial administration. Additionally, it was reported that dysregulated metabolites of tryptophan and activated indoleamine 2,3-dioxygenase (IDO) by inflammation were crucial parts of depression and aging regulated by the gut-brain axis.^402^ Supplementing mitochondria decreased the IDO activity and attenuated anxiety- and depression-like behaviors, highlighting the potential to treat psychiatric disorders.^397^

#### NAFLD and hepatic injuries

Failure of mitochondrial adaptation, particularly respiratory and metabolic deficiency, is one of the core features in the progression to insulin resistance and NAFLD.^24,403,404^ However, fat deposits gradually vanished after mitochondrial transplantation, along with decreased serum transaminase activity. Accordingly, it was observed that levels of ROS and malondialdehyde (MDA) were significantly reduced after mitochondrial transplantation, whereas Glutathione (GSH) content and superoxide dismutase (SOD) activity increased.^405^ These results suggested that mitochondrial transplantation could improve serum lipid metabolism and reduce hepatocyte injury from HFD-induced oxidation. Mitochondrial transplantation was also proven to have positive effects on hepatic injuries induced by toxic chemicals. In carbon tetrachloride (CCl_4_) induced hepatic injury, free radicals are formed and initiate lipid peroxidation to disrupt membrane integrity, resulting in mitochondrial damage and mass loss.^406^ However, the administration of liver mitochondria from healthy mice eliminated free radicals, upregulated GSH metabolism, and restored OXPHOS function in CCl_4_-induced chronic hepatic injury and fibrosis.^407^ Interestingly, the molecular mechanism of mitochondrial protection seemed to be correlated with the unfolded protein response (UPR^mt^) pathway, as it activated a series of anti-oxidative enzymes and increased mitophagy to rebuild mitochondrial homeostasis.^407^ Consistent results were observed in acetaminophen (APAP) induced liver injury where administration of exogenous mitochondria reversed mitochondrial structure disruption, GSH loss, and serum levels of alanine aminotransferase (ALT) and aspartate aminotransferase (AST).^408^ These provide novel therapeutic strategies to enhance resistance in response to hepatic injuries.

#### Acute respiratory distress syndrome/acute lung injury (ARDS/ALI)

With decades of foreshadowing of cell-based therapy, considerable potential has been shown to rescue the disrupted alveolar-capillary barrier in ARDS/ALI.^409–412^ As a protective mechanism of cell-based therapy, cell-based mitochondrial transfer has been reported to increase ATP content, promote an anti-inflammatory phenotype, and restore mitochondrial respiration in ARDS/ALI.^413,414^ Moreover, mitochondrial restoration which benefited from MSCs also protected epithelial cells from apoptosis in asthma.^415,416^ In cigarette smoke and LPS-induced chronic obstructive pulmonary disease (COPD), mitochondrial administration inhibited ROS production and thus decreased cholinergic sensitivity of the epithelium, resulting in altered inflammatory progression. However, direct protection of injured epithelial cells is not the only mechanism of mitochondrial transplantation against various tissue fates. It was reported that phagocytosis of macrophages was enhanced by mitochondria derived from MSCs, which partially involved the mechanism of antibacterial effects in an *E. coli* Pneumonia-induced ARDS model.^417^ In addition, an adoptive transfer model promoted M2 phenotype alteration and the phagocytic capacity of macrophages to resist LPS-induced ARDS. This was potentially achieved by increased OXPHOS capacity of macrophages via mitochondrial transfer induced by MSC-derived EVs.^418^ These studies highlighted cell-dependent mitochondrial transfer as an extension of cell-based therapy.

#### Spinal cord injury (SCI)

Mitochondrial dysfunction is increasingly recognized as a pivotal factor in the neuronal death cascades characteristic of central nervous system injuries.^419^ Empirical evidence has been garnered from a study using a Sprague-Dawley rat model, wherein local mitochondrial transplantation into the distal end of the injured nerve led to notable enhancements in neurobehaviors, nerve electrophysiology, and muscle activities.^420^ The administrated mitochondria mitigated oxidative stress and upregulated neurotrophic factor expression in both the injured nerves and denervated muscles, concurrently augmenting the pool of muscular progenitor cells, and increasing total muscle weight. In a parallel investigation, intraparenchymal infusion of allogeneic soleus muscles mitochondria into injured spinal cords resulted in the recovery of locomotor and sensory functions in rats with a reduction in SCI-induced cellular apoptosis and inflammation.^421^ Additionally, bone marrow stromal cells (BMSCs) have been observed to confer protection against SCI, attributed to their capacity to transfer mitochondria to injured neurons via intercellular gap junctions.^422^ Further advancements include the development of an engineered mitochondrial compound specifically designed to target macrophages within the SCI region.^423^ This approach demonstrated efficacy in attenuating pro-inflammatory profiles in macrophages, both in vitro and in vivo, thereby enhancing tissue regeneration and bolstering functional recovery in SCI models.

#### Other diseases

Studies on other diseases also illustrated the protective effect of mitochondrial transplantation, ranging from wound healing capacity to multiple sclerosis, tendinopathy, and osteoarthritis.^424–427^ Mitochondria from platelets were reported to stimulate the angiogenic potential of MSCs, thus promoting wound healing and tissue repair in chronic muscle injury.^424^ The directly administered mitochondria enhanced the TCA cycle and fatty acid synthesis, which was previously reported to favor epithelial cell proliferation for angiogenesis.^428^ In an autoimmune encephalomyelitis (EAE) model, intracerebroventricular induction of neural stem cells (NSCs) actively transferred mitochondria into mononuclear phagocytes and astrocytes, thereby ameliorating EAE disability in mice.^425^ In addition, mitochondria from L6 cells restored the protein levels of Tenascin C (TNC) in collagenase-damaged Achilles tendons, and were involved in the tendon protection process, along with the inhibition of apoptotic proteins (BID, Bax, and Bcl-2) and inflammatory cytokines (TNF-α, IL-1β, and IL-6).^426^ Similarly, the progression of osteoarthritis induced by monosodium iodoacetate was significantly inhibited by mitochondria from the skeletal (L6) myoblast cell line, which was also supported by improved pain phenotypes.^427^ Intravenous administration of mtDNA and TFAM mRNA-containing MSC-EVs was effective in reducing mitochondrial damage and inflammatory response in acute renal injury. This was possibly due to the protection of mitochondria in stressed HK-2 cells by TFAM-induced mtDNA maintenance.^40^ Additionally, it was discovered that mitochondria-located circular RNA (circRNA) and steatohepatitis-associated circRNA ATP5B regulator (circRNA SCAR) could reduce ROS production and avoid fibroblast activation via mitochondrial permeability transition during NAFLD.^429^ Through binding with ATP5B of the mPTP complex, the circRNA SCAR could block cyclophilin D-mPTP interactions, thereby inhibiting the opening of mPTP and reducing ROS production. Importantly, a decrease in ROS level and cytokine secretion was observed in circRNA SCAR-treated mice. The intravenous injection of mitochondria-targeting circRNA, which contained circRNA SCAR overexpression vectors, also evidenced the improvement of glucose and insulin resistance 2, along with weight loss and macrophage infiltration. These preclinical studies have broadened the applications of mitochondrial transplantation in non-ischemic diseases.

## Conclusion and perspective

Mitochondrial medicine has unfolded a remarkable trajectory of advancement across the span of several decades. Historically, mitochondrial diseases were predominantly ascribed to genetic aberrations, however, the current understanding has broadened to incorporate many common pathologies, as critical contributors.^1^ This paradigm shift intensified the appeal of developing therapeutic strategies targeting mitochondria. This review underscores the significance of tailored dietary supplements and adjustments, particularly the administration of essential nutrients such as carnitine, CoQ, creatine, and vitamin B2, alongside the potential benefits of ketogenic diets in leveraging alternative energy sources through ketone bodies, aiming at mitigating the metabolic intricacies inherent in mitochondrial pathologies. In addition, conventional pharmacological targets - inclusive of those implicated in regulating mitochondrial stress responses, metabolite pools, biogenesis, and dynamics- are transitioning to rigorous evaluation in randomized clinical trials, which represent a strategic pivot towards the dual roles of mitochondria in energy production and cellular signaling. Concurrently, the necessity for the conception and implementation of highly precise therapies becomes increasingly evident. Small molecules that are intricately designed to target membrane potential and cardiolipin of mitochondria were included at the inception stages of drug design, with the objective of augmenting mitochondrial delivery.^430^ Nonetheless, the journey from bench to bedside is fraught with challenges, including the clinical heterogeneity of mitochondrial disorders and the potential side effects of long-term dietary and pharmacological interventions. For instance, the protective role of ketogenic diets, while beneficial in simulating a fasting-like metabolic state, raises concerns regarding nutritional balance and sustainability, not to mention the observed risk of cardiac fibrosis in animal models. Similarly, the clinical efficacy of antioxidants and compounds targeting mitochondrial dynamics necessitates a careful balance, avoiding interference with physiological ROS signaling mechanisms essential for cellular homeostasis.

Mitochondrial transplantation is another strategy burgeoning on the horizon of therapeutic innovation. This involves the isolation of active mitochondria from individuals devoid of mitochondrial defects, followed by their introduction into compromised cells. Recently, Hayashida, K. et al. conducted a systematic review of pertinent evidence from both animal and human studies concerning mitochondrial transplantation in IR injury, offering a practical assessment of the categorization, concentration, and accessibility of data within this field.^431^ Twenty animal and two human studies were included, with 14 studies (approximately 70%) focusing on IR models in heart and brain tissues with high energy demand and metabolism. The collective findings from animal studies endorsed the ability of mitochondrial transplantation to mitigate IR injury, although these results were inconsistent concerning specific biomarkers and pathological alterations.^377,393,414,431^ These exogenous mitochondria are postulated to integrate with the existing functional networks within the recipient cells.^432^ However, symbiosis was hardly observed between exogenous and endogenous mitochondria, which might explain why some studies consider exogenous mitochondrial function as a substitution for endogenous damaged mitochondria.^376^ While most studies reported favorable results after mitochondrial transplantation, elucidation of the underlying mechanisms pertinent to its clinical translation remains an open challenge, including risks such as susceptibility during mitochondrial isolation, immune adaptation, endocytosis, and lysosome escape.^431,433^ The potential for xenotransplantation, deliberation over the most suitable source of mitochondria-whether autologous, allogenic, or xenogeneic, is still governed by a matrix of factors encompassing immunological compatibility, ethical implications, and the nuanced demands of clinical application. Autologous mitochondria, procured from the patient’s own tissues, emerge as the frontrunner in terms of biocompatibility, significantly reducing the risk of immune rejection and sidestepping the ethical complexities tethered to xenogeneic sources. However, this approach is not without its limitations, particularly in the context of systemic or congenital mitochondrial disorders where the availability of functionally intact mitochondria is often compromised.^434^ Allogenic mitochondria, sourced from human donors, represent a pragmatic alternative, particularly in acute settings where the requirement for fresh mitochondria may be increased due to the limited intervention window of opportunity.^388^ Xenogeneic mitochondria, while presenting an abundant and diverse reservoir, are currently confined to the experimental realm, predominantly due to heightened immunogenic risks and profound ethical considerations. Recently, of great concern to researchers, a nanothylakoid unit (NTU) based on the controllable photosynthetic system was developed as cross-species ATP and NADPH providers to treat osteoarthritis.^435^ Plant-derived NTUs were extruded and encapsulated by the purified chondrocyte membrane (CM-NTUs) to avoid immune rejection. According to proteomic analysis, these chondrocyte membrane proteins were highly correlated with vesicle targeting and membrane fusion, which might result in the efficient uptake by chondrocytes rather than being eliminated by macrophages or intracellular lysosomes. In addition, proteomic profiling demonstrated that mitochondrial diversity reveals heterogeneity in morphology, state, function, and diseases, leaving a large mitochondrial pool to be discussed.^436^ For example, in brown adipocytes, PDMs are inactive in fusion-fission dynamics but favored with increased ATP synthesis and lipid expansion capacity, compared to cytoplasmic mitochondria.^437^ Stem cells might also be universal mitochondrial donors, as mitochondrial transfer partially participated in the therapeutic effect of MSCs.^438^ Viable transplanted mitochondria seemed to be indispensable to the postischemic restoration whereas deactivated mitochondria, such as frozen mitochondria, mitochondrial genomes, and ATP-containing vesicles, did not achieve the same results.^43,372^ To shorten the preparation time, McCully, J. D. et al. proposed a tissue pool to obtain fresh mitochondria, including pectoralis major muscle, rectus abdominis or even sternocleidomastoid muscle.^439^ Comprehensive mechanistic investigations and more extensive preclinical studies remain essential.

The mitochondrial transplantation process largely involves the internalization of transplanted mitochondria at the cell membrane level to mitigate the biological information in the cell. However, the molecular mechanism of how the mitochondria can be integrated into endogenous mitochondrial network remain heterogenous and even largely unknown. Several studies have suggested versatile mechanisms may be involved in mitochondrial horizontal transfer between cells. In vitro data showed that the autologous driving force of mitochondrial uptake included endocytosis of recipient cells, such as macropinocytosis, clathrin-mediated endocytosis, or actin polymerization, which vary among different mitochondrial recipients.^440,441^ Non-specific macropinocytosis was also involved in some cases, evidenced by the specific inhibitor of micropinocytosis, ethyl isopropyl amiloride (EIPA).^85^ Endocytosis is a cellular process whereby a vesicle engulfs extracellular material, and it was suggested that transplanted mitochondria can be internalized through this process.^387,424,441^ In addition, receptor, actin, and ATP-dependent phagocytosis was validated to participate in engulfment of extracellular mitochondria via membrane invagination.^442^ Gap junctions, which are intercellular channels that allow for the exchange of small molecules, have also been implicated in mitochondrial internalization.^413,422,443^ Tunneling nanotubes (TNT) are highly sensitive nanotube structures that allow for direct communication between cells and have long been implicated in mitochondrial transfer.^417,444^ Partial cell fusion is another mechanism that has been proposed, involving the direct fusion between cells and mitochondria-sharing after TNT was inhibited.^445^ Recent studies also suggested that fusogenic syncytins on mitochondria, along with their receptors, might facilitate the mitochondrial internalization process under cancerous circumstances.^446^ However, current evidence points out single forms of internalization, as transplantation efficiency appears to be hyperresponsive to single inhibitors.^415,422,424,441^ Additionally, the employment of mitochondrial dyes in assessing mitochondrial incorporation during transplantation, while a common practice in both in vitro and in vivo studies, is hindered by the propensity of these dyes to leak.^447^ This leakage can lead to non-specific staining and false-positive results, as these mitochondrial dyes may escape from both donor and recipient cells, inadvertently staining neighboring mitochondria. The optimal approach for studying mitochondrial transfer in both in vitro and in vivo settings involves the use of stable transgenes encoding mitochondrially localized tags or fluorescent proteins. This mitochondrial reporter system, adaptable for lineage-specific mitochondrial reporter mice or for broad cell labeling, offers stable and precise tracking of mitochondria in recipient cells. However, a major concern is to avoid misinterpretation due to unintended Cre activity in other cell types when establishing stringent cell-type-specific transgene expression based on Cre recombinase systems.^447^ Collectively, a better understanding of these internalization mechanisms will help optimize the efficacy and safety of mitochondrial transplantation for various clinical applications.

The future direction of mitochondrial dysfunction therapeutics likely resides in personalized medicine, wherein genetic and metabolic profiling could tailor interventions to individual patient needs, enhancing both efficacy and safety. As the therapeutic landscape for mitochondrial dysfunction continues to mature, the integration of dietary, pharmacological, and preventive strategies holds the promise of a more comprehensive and effective approach to managing these complex diseases. The endeavor to translate these advances into clinical practice underscores the need for a multidisciplinary effort, bridging the gap between molecular insights and therapeutic innovation to forge a path towards improved patient outcomes in the realm of mitochondrial pathology.
